# Enantioselective PCCP Brønsted acid-catalyzed aza-Piancatelli rearrangement

**DOI:** 10.3762/bjoc.15.160

**Published:** 2019-07-12

**Authors:** Gabrielle R Hammersley, Meghan F Nichol, Helena C Steffens, Jose M Delgado, Gesine K Veits, Javier Read de Alaniz

**Affiliations:** 1 Department of Chemistry and Biochemistry, University of California Santa Barbara, Santa Barbara, CA 93106-9510, USA

**Keywords:** aza-Piancatelli, Brønsted acid, cyclopentane, furylcarbinols, PCCP

## Abstract

An enantioselective aza-Piancatelli rearrangement has been developed using a chiral Brønsted acid based on pentacarboxycyclopentadiene (PCCP). This reaction provides rapid access to valuable chiral 4-amino-2-cyclopentenone building blocks from readily available starting material and is operationally simple.

## Introduction

The discovery of a wide range of cyclopentane containing natural products [1–2] and biologically active molecules such as palau’amine [3–4], agelastatin A [5–7], pactamycin [8–10], and aristeromycin [11–12], has created a demand for new methodologies to construct this privileged scaffold. The aza-Piancatelli reaction has recently emerged as a particularly attractive method to access densely functionalized cyclopentene cores bearing nitrogen substituents directly from readily available 2-furylcarbinols [13–15]. Inspired by Piancatelli’s original work in 1976 [16], our group has developed both the inter- and intramolecular aza-Piancatelli reaction using commercially available dysprosium trifluoromethanesulfonate (Dy(OTf)_3_) as a catalyst with a range of nitrogen nucleophiles [17–23]. The range of catalytic systems facilitating this reaction has been extended to include other Brønsted and Lewis acids, such as phosphomolybdic acid (PMA) [24], Ca(NTf_2_)_2_ [25], In(OTf)_3_ [26], La(OTf)_3_ [27], and BF_3_·OEt_2_ [28], as well as other nucleophiles [29–31].

**Figure 1:**
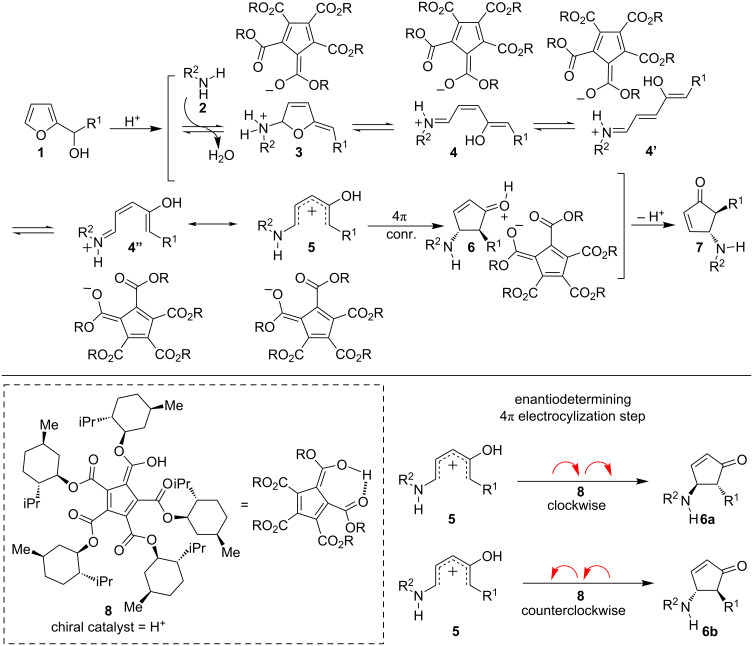
Proposed mechanism of the asymmetric aza-Piancatelli reaction.

In all cases examined, the products of the aza-Piancatelli reaction have a *trans* relationship between the C4 and C5 substituents [13–15]. It is believed that the 4π conrotatory electrocyclization step that converts the pentadienyl cation **5** into the corresponding cyclopentenone adduct **6** is responsible for controlling the relative diastereoselectivity in this cascade rearrangement (Figure 1). Analogous to the Nazarov cyclization, controlling the absolute stereochemistry can be achieved by governing the direction of the conrotatory electrocyclization, clockwise vs counterclockwise [32–34]. Despite the direct relationship to the asymmetric Nazarov cyclization, however, it was not until 2016 that the first asymmetric aza-Piancatelli reaction was described. To control the absolute stereochemistry of the aza-Piancatelli rearrangement, Rueping [35], Sun [36], and Patil [37] independently demonstrated that chiral phosphoric acids can be used as an enantioselectivity-inducing element capable of controlling the clockwise or counterclockwise conrotation of the key 4π electrocyclization step.

Although the utility of these asymmetric catalytic systems is unquestionable, the ability to identify the optimal catalyst is not straightforward. In each case, extensive optimization of the reaction conditions was required to achieve high enantioselectivity and good yield, with small variations to the catalyst architecture or solvent having dramatic effects on enantioselectivity or yield. Because of these challenges and our group’s ongoing interest in further developing the aza-Piancatelli reaction, we sought to identify other asymmetric catalytic systems capable of controlling the absolute stereochemistry. To this end, we envisioned that the chiral pentacarboxycyclopentadiene (PCCP) Brønsted acid catalyst (**8**) recently developed by the Lambert lab might be suitable (Figure 1) [38]. First, the p*K*_a_ values measured in acetonitrile (MeCN) are lower than chiral phosphoric acids (Brønsted acid p*K*_a_ = 8.85 vs chiral phosphoric acids p*K*_a_ = 12–14) [38]. Given the enhanced acidity, we reasoned that this type of chiral Brønsted acid catalyst could facilitate the dehydration reaction of the furylcarbinol to generate the oxocarbenium intermediate. Second, by analogy to asymmetric induction in aza-Piancatelli reactions with chiral phosphoric acids, where enantioselectivity has generally been achieved by strategically installing bulky groups on the hydrogen-bonding catalysts, we hypothesized that a PCCP-derived catalyst could be used. It is proposed that the chiral anion moieties serve to interact with the key intermediate **5** during the enantiodetermining electrocyclization step [35–37]. A tentative mechanistic hypothesis for the asymmetric aza-Piancatelli is shown in Figure 1. Finally, this new catalyst can be produced inexpensively and on scale, features that are attractive for developing a wide range of asymmetric transformations.

## Results and Discussion

Herein, we describe our initial efforts in this area using chiral Brønsted acid catalyst **8**. Our investigations began by examining the reaction of *para*-iodoaniline (**2a**) with furylcarbinol **1a** in dichloromethane (DCM) in the presence of 5 mol % chiral Brønsted acid **8** (Table 1). We were pleased to find that **8** catalyzed the desired asymmetric transformation at 40 °C, affording 4-aminocyclopentenone **7a** in 78% yield and a moderate 65% ee. At lower temperatures (30 °C and 23 °C (rt)) the selectivity increased to 73% and 78% ee, respectively. For consistency the optimization studies were all allowed to run for 48 h with higher temperatures being more efficient (Table 1, entries 1–3). Importantly, the catalytic activity did not diminish with extended reaction time and the yield of the room temperature reaction could be increased to 70% by extending the reaction time from 48 h to 120 h (Table 1, entry 4). The asymmetric reaction was found to be most effective at 23 °C (Table 1, entries 3 and 4). Unfortunately, attempts to lower the temperature further resulted in exceedingly long reaction times, greater than 5 days, and thus were not pursued. Next the effects of aromatic and halogenated solvents were evaluated (Table 1, entries 5–7), with DCM proving optimal. We developed the reaction using 5 mol % catalyst in DCM at 23 °C as the optimized reaction conditions.

**Table 1:** Initial optimization studies.

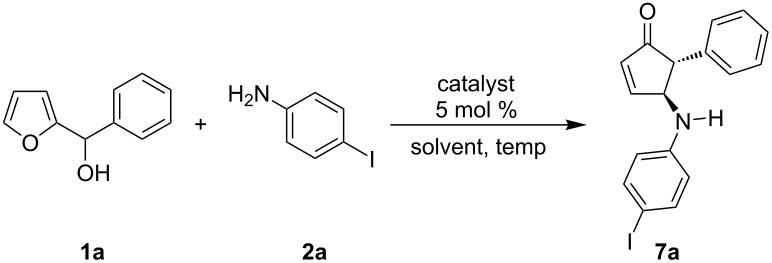				

Entry	Solvent	Temp (°C)	ee (%)	Yield (%)

1	DCM	40	65	78
2	DCM	30	73	46
3	DCM	rt	78	26
4^a^	DCM	rt	75	70
5	DCE	rt	68	23
6	fluorobenzene	rt	76	20
7	toluene	rt	75	12

^a^This reaction was run for 5 days whereas the rest of the reactions were run for 2 days. Dichloromethane (DCM), 1,2-dichloroethane (DCE), room temperature (rt).

**Scheme 1:**
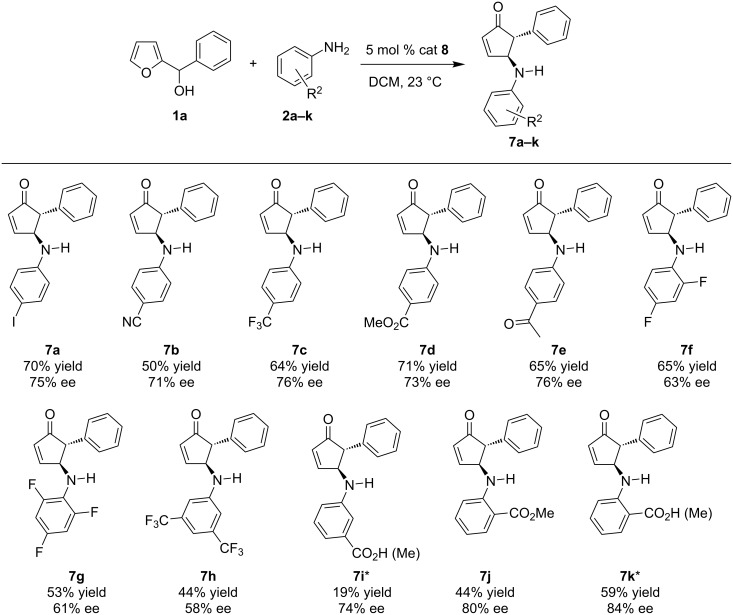
Asymmetric aza-Piancatelli rearrangement with a range of substituted anilines. *To simplify the purification process, carboxylic acids were transformed in situ into the corresponding methyl ester using (trimethylsilyl)diazomethane.

With optimized reaction conditions in hand, we set out to explore the scope and limitations of the asymmetric reaction (Scheme 1). Initially, we examined the reaction of various anilines with furylcarbinol **1a**. Scheme 1 summarizes the results obtained with *ortho-, meta-,* and *para-*substituted aniline derivatives. The reaction of anilines bearing an electron-withdrawing group at the *para-*position afforded the optimal balance between efficiency and enantioselectivity. Consistent with Rueping’s work [35], *ortho-*aminobenzoic acid, which contains an additional hydrogen bond group afforded the best selectivity (**7k**). To simplify the purification process, all acid products were transformed into the corresponding methyl ester in situ using (trimethylsilyl)diazomethane. A slight drop in enantioselectivity is observed when the benzoic acid group was moved to the *meta-*position or when using the methyl ester (**7i** and **7j**)**.** The absolute stereochemistry of the product **7k** was assigned by comparison to literature [35] and the other products were assigned by analogy.

**Scheme 2:**
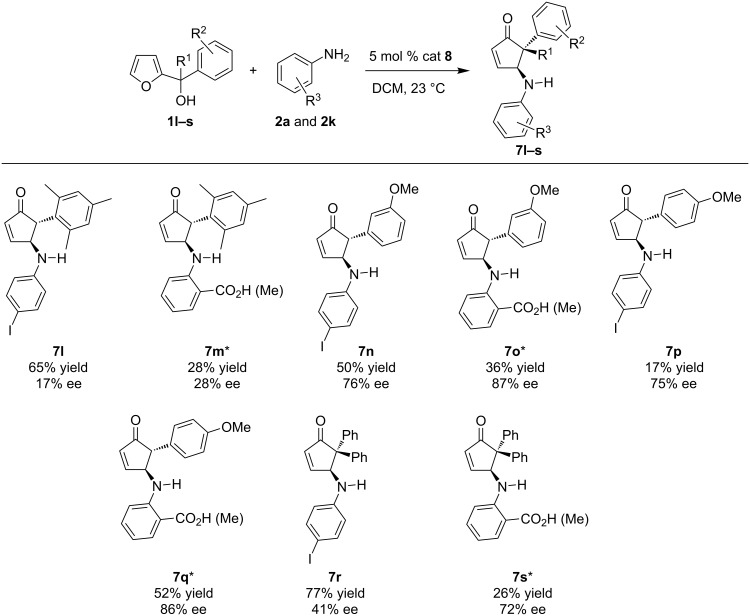
Asymmetric aza-Piancatelli rearrangement with a range of substituted furylcarbinols. *To simplify the purification process, carboxylic acids were transformed in situ into the corresponding methyl ester using (trimethylsilyl)diazomethane.

To further expand the substrate scope, we investigated the effects on both reactivity and enantioselectivity when substituted furylcarbinols were used with either *para-*iodoaniline (**2a**) or *ortho*-aminobenzoic acid (**2k**, Scheme 2). In every case examined, the *ortho*-aminobenzoic acid gave higher selectivity compared to the corresponding *para*-iodoaniline, which supports the importance of the additional hydrogen bonding capability of the carboxylic acid group. In contrast, with the exception of **7p** and **7q**, a lower yield was obtained when *ortho-*aminobenzoic acid was used. Presumably, this decrease in efficiency is due to the increased steric bulk on the aniline, which slows the initial nucleophilic attack on the furan ring required to initiate the cascade sequence. This highlights that a balance between nucleophilicity and hydrogen bond capabilities are required to obtain an efficient and selective reaction. Compared to the rearrangement of furylcarbinol **1a**, sterically bulky aryl groups attached to the furylcarbinol resulted in significantly diminished enantioselectivity (**7l** and **7m**). In contrast, placing a substituent at either the *meta*- or *para-*position showed no effect on the selectivity when compared to an unsubstituted phenyl group. For example, **1a** afforded the desired product in 75% ee, while **1n** and **1p** afforded the cyclopentenone product in 76% ee and 75% ee, respectively. In general, the enantioselectivity of the reactions with *para*-iodoaniline and *ortho-*aminobenzoic acid behaved similarly, with the selectivity being influenced most obviously by changes in the furylcarbinol. However, a dramatic difference in selectivity was observed when employing a tertiary furylcarbinol (**7r** and **7s**). In this case, *ortho*-aminobenzoic acid resulted in the desired product with good enantioselectivity (72% ee), while *para*-iodoaniline only gave a modest 41% ee. The exact nature for the decrease in enantioselectivity in this case with *para*-iodoaniline is not clear.

## Conclusion

In summary, we have developed an efficient asymmetric aza-Piancatelli rearrangement that constructs a carbon–carbon bond plus a carbon–nitrogen bond and controls the absolute stereochemistry of the two stereocenters, through control of the direction of conrotation, in a single operation. This strategy offers a practical alternative to using chiral phosphoric acids and demonstrates that chiral PCCP-based Brønsted acid catalysts can be used to control the absolute stereochemistry of the aza-Piancatelli rearrangement. The PCCP chiral Brønsted acid catalyzed reaction shows good substrate scope and proceeds well with a range of aniline and furylcarbinol derivatives. The ability to control the absolute stereochemistry of the 4π electrocylization using an inexpensive and easy to prepare chiral Brønsted acid catalyst holds tremendous promise for the aza-Piancatelli and related rearrangements.

## Experimental

**General procedure for the rearrangement:** Furylcarbinol **1** and aniline **2** were dissolved in DCM. At 23 °C, 5 mol % of the catalyst **8** was added to the reaction mixture and the reaction mixture was stirred for 5 days. The reaction was then quenched with saturated aqueous sodium bicarbonate and extracted with DCM (3 × 5 mL). The combined organic layers were dried over MgSO_4_, filtered, and concentrated in vacuo. The residue was then purified by column chromatography to afford cyclopentenone **7**.

## Supporting Information

File 1 Experimental part and copies of NMR spectra.
