# Supplementary material for: Enantioselective PCCP Brønsted acid-catalyzed aza-Piancatelli rearrangement
Source: Beilstein J Org Chem. 2019 Jul 12;15:1569–74. doi: 10.3762/bjoc.15.160 (PMC6633596; doi:10.3762/bjoc.15.160)

BEILSTEIN JOURNAL OF ORGANIC CHEMISTRY

Supporting Information

for

Enantioselective PCCP Brønsted acid-catalyzed aza-Piancatelli rearrangement

Gabrielle R. Hammersley, Meghan F. Nichol, Helena C. Steffens, Jose M. Delgado, Gesine K. Veits and Javier Read de Alaniz

Beilstein J. Org. Chem. **2019,** *15,* 1569–1574. doi:10.3762/bjoc.15.160

Experimental part and copies of NMR spectra

License and Terms: This is a supporting information file under the terms of the Creative Commons Attribution License (http://creativecommons.org/licenses/by/4.0). Please note that the reuse, redistribution and reproduction in particular requires that the authors and source are credited.
The license is subject to the *Beilstein Journal of Organic Chemistry* terms and conditions: (https://www.beilstein-journals.org/bjoc)

Table of Contents

Materials and methods S2

Experimental procedures and data: S3

References S14

NMR Spectra S15

Materials and methods

Unless stated otherwise, reactions were conducted in oven dried glassware under an atmosphere of N_2 using reagent grade solvents. All commercially obtained reagents were used as received. Reaction temperatures were controlled using a Heidolph temperature modulator, and unless otherwise, reactions were performed at room temperature (rt, approximately 23 °C). Thin-layer chromatography (TLC) was conducted with E. Merck silica gel 60 F254 pre-coated plates (0.25 mm) and visualized by exposure to UV light (254 nm) or stained with potassium permanganate or anisaldehyde. Flash chromatography was preformed using normal phase silica gel (60 Å, 230–240 mesh, Geduran®) ^1H NMR spectra were recorded on Varian Spectrometers (at 400, 500, and 600 MHz) and are reported relative to deuterated solvent signals. Data for ^1H NMR spectra are reported as follows: chemical shift (δ ppm), multiplicity, coupling constant (Hz) and integration. ^{13}C NMR spectra were recorded on Varian spectrometers (at 100, 125, and 150 MHz). Data for ^{13}C NMR spectra are reported in terms of chemical shift. IR spectra were recorded on a Perkin Elmer Spectrum 100 FTIR and a Bruker Alpha FTIR and are reported in terms of frequency of absorption (cm^{-1}). High resolution mass spectra were obtained from the UC Santa Barbara Mass Spectrometry Facility. Enantiomeric excess was determined by use of a Shimadzu Prominence high-performance liquid chromatography (HPLC) system. Furylcarbinols **1a**, **1b**, **1c**, **1d**, and **1e** were prepared according to literature precedent by reacting furfural with the corresponding Grignard reagent.[1] The PCCP catalyst **8** was prepared according to literature precedent.[2]

Experimental procedures and data:

General procedures:

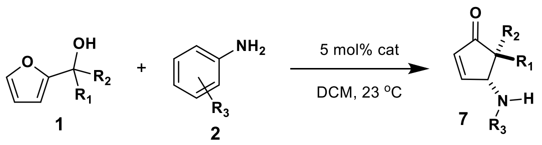

General procedure for the rearrangement: Furylcarbinol **1** and aniline **2** were dissolved in DCM. At 23 °C, 5 mol % of the catalyst was added to the reaction mixture. Once capped, the reaction was allowed to stir for 5 days. The reaction was then quenched with saturated aqueous sodium bicarbonate and extracted with DCM (3 × 5 mL). The combined organic layers were dried over $MgSO_4$, filtered, and concentrated in vacuo. The residue was then purified via column chromatography to afford the cyclopentenone **7**.

Synthesis procedures:

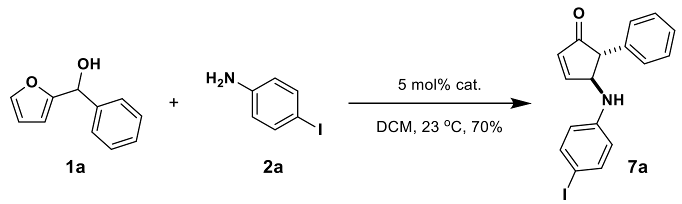

(4*S*,5*R*)-4-((4-Iodophenyl)amino)-5-phenylcyclopent-2-en-1-one (7a): According to the general procedure, the catalyst **8** (4.7 mg, 0.005 mmol, 0.05 equiv) was added to furan-2-yl(phenyl)methanol **1a** (20.0 mg, 0.11 mmol, 1.2 equiv) and 4-iodoaniline (**2a**, 21.0 mg, 0.10 mmol, 1.0 equiv) in 1 mL of anhydrous DCM. The resulting reaction mixture was allowed to stir at room temperature for 5 days before being quenched with 5 mL of saturated aqueous sodium bicarbonate and extracted with DCM (3 × 5 mL). The combined organic layers were dried over $MgSO_4$, filtered, and concentrated in vacuo. The residue was purified via column chromatography to afford cyclopentenone **7a** (25.1 mg, 70%) as a solid. Spectral data matches literature values[3]; The enantiomeric purity of the product determined by HPLC: 75% ee (Chiralpak IB column, *n*-hexane/iPrOH = 90:10, flow rate 1.0 mL/min, λ = 254 nm), t_r (minor) = 19.5 min, t_r (major) = 25.0 min; $[\alpha]^{rt}_D$ = 87.4 (*c* = 0.53 in CH_2Cl_2).

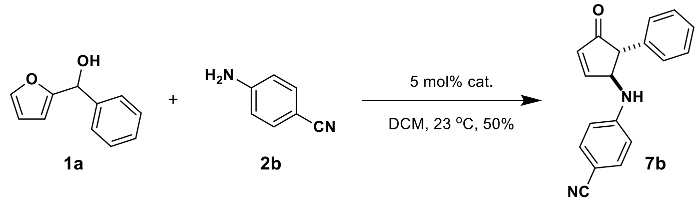

4-(((1*S*,5*R*)-4-Oxo-5-phenylcyclopent-2-en-1-yl)amino)benzonitrile (7b): According to the general procedure, the catalyst **8** (4.7 mg, 0.005 mmol, 0.05 equiv) was added to **1a** (20.0 mg, 0.11 mmol, 1.2 equiv) and 4-aminobenzonitrile (**2b**, 11.3 mg, 0.10 mmol, 1.0 equiv) in 1 mL of anhydrous DCM. The resulting reaction mixture was allowed to stir at room temperature for 5 days before being quenched with 5 mL of saturated aqueous sodium bicarbonate and extracted with DCM (3 × 5 mL). The combined organic layers were dried over $MgSO_4$, filtered, and concentrated in vacuo. The residue was purified via column chromatography to afford cyclopentenone **7b** (13.1 mg, 50%) as a solid. Spectral data matches literature values[4]; The enantiomeric purity of the product determined by HPLC: 71% ee (Chiralpak IB column, *n*-hexane/iPrOH = 90:10, flow rate 1.0 mL/min, λ = 254 nm), t_r (minor) = 30.2 min, t_r (major) = 38.4 min; $[\alpha]^{rt}_D$ = 87.0 (*c* = 0.53 in CH_2Cl_2).

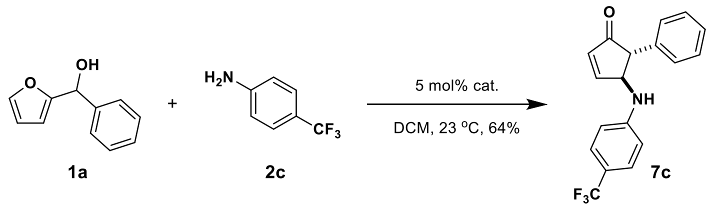

(4*S*,5*R*)-5-Phenyl-4-((4-(trifluoromethyl)phenyl)amino)cyclopent-2-en-1-one (7c): According to the general procedure, the catalyst **8** (4.7 mg, 0.005 mmol, 0.05 equiv) was added to **1a** (20.0 mg, 0.11 mmol, 1.2 equiv) and 4-(trifluoromethyl)aniline (**2c**, 12 μL, 0.10 mmol, 1.0 equiv) in 1 mL of anhydrous DCM. The resulting reaction mixture was allowed to stir at room temperature for 5 days before being quenched with 5 mL of saturated aqueous sodium bicarbonate and extracted with DCM (3 × 5 mL). The combined organic layers were dried over $MgSO_4$, filtered, and concentrated in vacuo. The residue was purified via column chromatography to afford cyclopentenone **7c** (19.4 mg, 64%) as a solid. Spectral data matches literature values[5]; The enantiomeric purity of the product determined by HPLC: 76% ee (Chiralpak IB column, *n*-hexane/iPrOH = 90:10, flow rate 1.0 mL/min, λ = 254 nm), t_r (minor) = 15.0 min, t_r (major) = 19.3 min; $[\alpha]^{rt}_D$ = 30.5 (*c* = 0.24 in CH_2Cl_2).

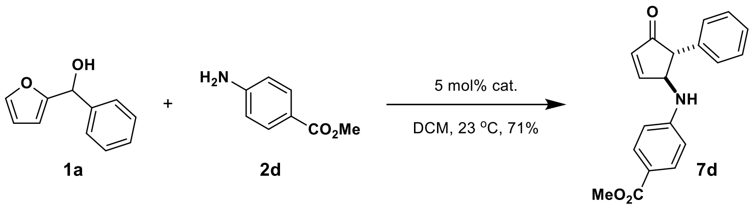

Methyl 4-(((1*S*,5*R*)-4-oxo-5-phenylcyclopent-2-en-1-yl)amino)benzoate (7d): According to the general procedure, the catalyst **8** (4.7 mg, 0.005 mmol, 0.05 equiv) was

added to **1a** (20.0 mg, 0.11 mmol, 1.2 equiv) and methyl 4-aminobenzoate (**2d**, 14.5 mg, 0.10 mmol, 1.0 equiv) in 1 mL of anhydrous DCM. The resulting reaction mixture was allowed to stir at room temperature for 5 days before being quenched with 5 mL of saturated aqueous sodium bicarbonate and extracted with DCM (3 × 5 mL). The combined organic layers were dried over $MgSO_4$, filtered, and concentrated in vacuo. The residue was purified via column chromatography to afford cyclopentenone **7d** (20.9 mg, 71%) as a solid. Spectral data matches literature values[3]; The enantiomeric purity of the product determined by HPLC: 73% ee (Chiralpak IB column, *n*-hexane/iPrOH = 90:10, flow rate 1.0 mL/min, λ = 254 nm), t_r (minor) = 27.0 min, t_r (major) = 31.9 min; $[\alpha]^{rt}_D$ = 76.6 (c = 0.50 in CH_2Cl_2).

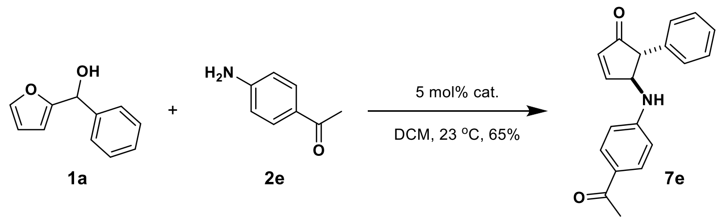

(4*S*,5*R*)-4-((4-Acetylphenyl)amino)-5-phenylcyclopent-2-en-1-one (7e): According to the general procedure, the catalyst **8** (4.7mg, 0.005 mmol, 0.05 equiv) was added to **1a** (20.0 mg, 0.11 mmol, 1.2 equiv) and 4-aminoacetophenone (**2e**, 12.9 mg, 0.10 mmol, 1.0 equiv) in 1 mL of anhydrous DCM. The resulting reaction mixture was allowed to stir at room temperature for 5 days before being quenched with 5 mL of saturated aqueous sodium bicarbonate and extracted with DCM (3 × 5 mL). The combined organic layers were dried over $MgSO_4$, filtered, and concentrated in vacuo. The residue was purified via column chromatography to afford cyclopentenone **7e** (18.1 mg, 65%) as a solid. ^1H NMR (500 MHz, Chloroform-*d*) δ 7.79 – 7.74 (m, 3H), 7.40 – 7.29 (m, 3H), 7.14 (dt, J = 7.7, 1.6 Hz, 2H), 6.52 – 6.45 (m, 3H), 4.84 (t, J = 2.2 Hz, 1H), 4.54 (s, 1H), 3.41 (q, J = 2.3 Hz, 1H), 2.48 (d, J = 1.9 Hz, 3H); ^{13}C NMR (125 MHz, Chloroform-*d*) δ 196.4, 160.6, 150.3, 137.7, 135.6, 130.9, 129.3, 128.2, 128.0, 127.8, 112.6, 62.8, 60.4, 26.2; IR 3328, 3068, 3000, 2921, 1713, 1648, 1569, 1525, 1339, 1275, 1165, 824, 700, 532 cm^{-1}; HRMS (ESI), calculated for $C_{19}H_{17}NO_2$: (M^+) 291.1259; observed 291.1270; The enantiomeric purity of the product determined by HPLC: 76% ee (Chiralpak IB column, *n*-hexane/iPrOH = 90:10, flow rate 1.0 mL/min, λ = 254 nm), t_r (minor) = 39.3 min, t_r (major) = 43.8 min; $[\alpha]^{rt}_D$ = 76.0 (c = 0.51 in CH_2Cl_2).

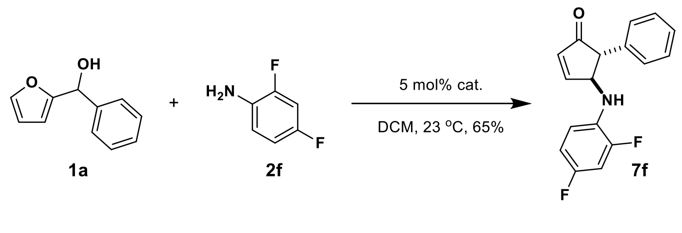

(4*S*,5*R*)-4-((2,4-Difluorophenyl)amino)-5-phenylcyclopent-2-en-1-one (7f): According to the general procedure, the catalyst **8** (4.7 mg, 0.005 mmol, 0.05 equiv) was added to **1a** (20.0 mg, 0.11 mmol, 1.2 equiv) and 2,4-difluoroaniline (**2f**, 9.7 µL, 0.10 mmol, 1.0 equiv) in 1 mL of anhydrous DCM. The resulting reaction mixture was allowed to stir at room temperature for 5 days before being quenched with 5 mL of saturated aqueous sodium bicarbonate and extracted with DCM (3 × 5 mL). The combined organic layers were dried over $MgSO_4$, filtered, and concentrated in vacuo. The residue was purified via column chromatography to afford cyclopentenone **7f** (17.7 mg, 65%) as a solid. ^{1}H NMR (500 MHz, Chloroform-*d*) δ 7.76 (dd, *J* = 5.7, 2.3 Hz, 1H), 7.38 – 7.27 (m, 3H), 7.14 – 7.09 (m, 2H), 6.80 (ddd, *J* = 11.3, 8.4, 2.8 Hz, 1H), 6.63 – 6.57 (m, 1H), 6.45 (dd, *J* = 5.7, 1.7 Hz, 1H), 6.38 (td, *J* = 9.2, 5.3 Hz, 1H), 4.72 (q, *J* = 2.3 Hz, 1H), 4.03 (s, 1H), 3.38 (d, *J* = 2.6 Hz, 1H); ^{13}C NMR (125 MHz, Chloroform-*d*) δ 206.1, 161.1, 156.1, 137.9, 135.3, 129.6, 129.2, 128.7, 128.7, 128.1, 127.7, 114.1, 114.0, 114.0, 114.0, 111.0, 111.0, 110.9, 110.9, 105.1, 104.2, 104.0, 104.0, 103.8, 63.8, 60.4; IR 3374, 3063, 3029, 2919, 1703, 1600, 1516, 1431, 1265, 1142, 959, 698 cm^{-1}; HRMS (ESI), calculated for $C_{17}H_{13}F_2NO$: (M^+) 285.0965; observed 285.0961; The enantiomeric purity of the product determined by HPLC: 63% ee (Chiralpak IB column, *n*-hexane/iPrOH = 90:10, flow rate 1.0 mL/min, λ = 254 nm), t_r (minor) = 13.4 min, t_r (major) = 18.0 min; $[\alpha]^{rt}_D$ = 76.8 (*c* = 0.47 in CH_2Cl_2).

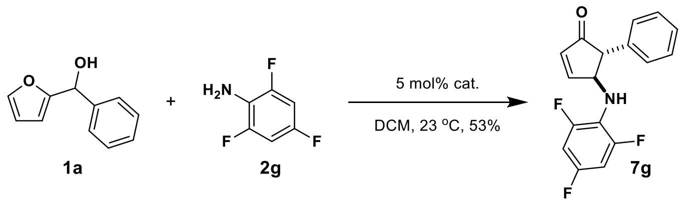

(4*S*,5*R*)-5-Phenyl-4-((2,4,6-trifluorophenyl)amino)cyclopent-2-en-1-one (7g): According to the general procedure, the catalyst **8** (4.7 mg, 0.005 mmol, 0.05 equiv) was added to **1a** (20.0 mg, 0.11 mmol, 1.2 equiv) and 2,4,6-trifluoroaniline (**2g**, 14.1 mg, 0.10 mmol, 1.0 equiv) in 1 mL of anhydrous DCM. The resulting reaction mixture was allowed to stir at room temperature for 5 days before being quenched with 5 mL of saturated aqueous sodium bicarbonate and extracted with DCM (3 × 5 mL). The combined organic layers were dried over $MgSO_4$, filtered, and concentrated in vacuo. The residue was purified via column chromatography to afford cyclopentenone **7g** (15.4 mg, 53%) as a solid. Spectral data matches literature values[3]; The enantiomeric purity of the product determined by HPLC: 61% ee (Chiralpak IB column, *n*-hexane/iPrOH = 90:10, flow rate 1.0 mL/min, λ = 254 nm), t_r (minor) = 12.6 min, t_r (major) = 14.7 min; $[\alpha]^{rt}_D$ = 97.4 (*c* = 0.50 in CH_2Cl_2).

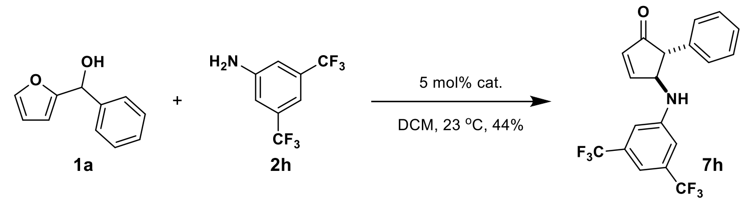

(4*S*,5*R*)-4-((3,5-Bis(trifluoromethyl)phenyl)amino)-5-phenylcyclopent-2-en-1-one (7h): According to the general procedure, the catalyst **8** (4.7 mg, 0.005 mmol, 0.05 equiv) was added to **1a** (20.0 mg, 0.11 mmol, 1.2 equiv) and 3,5-bis(trifluoromethyl)aniline (**2h**, 14.9 µL, 0.10 mmol, 1.0 equiv) in 1 mL of anhydrous DCM. The resulting reaction mixture was allowed to stir at room temperature for 5 days before being quenched with 5 mL of saturated aqueous sodium bicarbonate and extracted with DCM (3 × 5 mL). The combined organic layers were dried over $MgSO_4$, filtered, and concentrated in vacuo. The residue was purified via column chromatography to afford cyclopentenone **7h** (16.2 mg, 44%) as a solid. Spectral data matches literature values[3]; The enantiomeric purity of the product determined by HPLC: 58% ee (Chiralpak IB column, *n*-hexane/iPrOH = 90:10, flow rate 1.0 mL/min, λ = 254 nm), t_r (minor) = 20.4 min, t_r (major) = 24.3 min; $[\alpha]^{rt}_D$ = 14.7 (*c* = 1.10 in CH_2Cl_2).

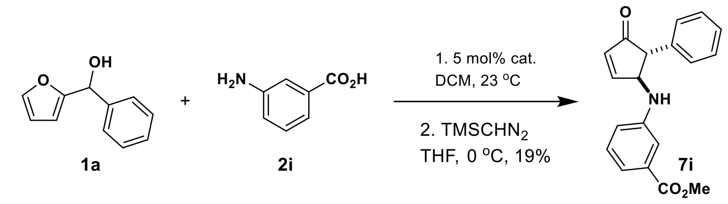

Methyl 3-(((1*S*,5*R*)-4-oxo-5-phenylcyclopent-2-en-1-yl)amino)benzoate (7i): According to the general procedure, the catalyst **8** (4.7 mg, 0.005 mmol, 0.05 equiv) was added to **1a** (20.0 mg, 0.11 mmol, 1.2 equiv) and 3-aminobenzoic acid (**2i**, 13.1 mg, 0.10 mmol, 1.0 equiv) in 1 mL of anhydrous DCM. The resulting reaction mixture was allowed to stir at room temperature for 5 days before being concentrated in vacuo. The residue is dissolved into 0.5 mL of anhydrous THF and cooled to 0 °C. (Trimethysilyl)diazomethane (2 M in hexanes; 0.19 mL, 0.38 mmol, 4.0 equiv) was added dropwise to the reaction and allowed to stir for 15 minutes before being quenched with MeOH. The reaction was then extracted with DCM and water (3 × 10 mL). The combined organic layers were dried over $MgSO_4$, filtered, and concentrated in vacuo. The residue was purified via column chromatography to afford cyclopentenone **7i** (5.6 mg, 19%) as a solid. ^{1}H NMR (500 MHz, Chloroform-*d*) δ 7.78 (dd, *J* = 5.8, 2.3 Hz, 1H), 7.43 – 7.40 (m, 1H), 7.38 – 7.28 (m, 3H), 7.21 – 7.12 (m, 4H), 6.70 (ddd, *J* = 8.2, 2.6, 1.0 Hz, 1H), 6.45 (dd, *J* = 5.8, 1.7 Hz, 1H), 4.81 (s, 1H), 4.11 (d, *J* = 8.7 Hz, 1H), 3.83 (s, 3H), 3.39 (d, *J* = 2.7 Hz, 1H); ^{13}C NMR (125 MHz, Chloroform-*d*) δ 206.2, 167.2, 161.2, 146.3, 137.9, 135.2, 131.5, 129.6, 129.2, 128.1, 127.6, 120.1, 118.3, 114.6, 105.2, 63.4, 60.4, 52.2; IR 3378, 3329, 3028, 2949, 1698, 1605, 1585, 1530, 1337, 1276, 1110, 744, 699, 545 cm^{-1}; HRMS (ESI), calculated for $C_{19}H_{17}NO_3$: (M^+) 307.1208; observed 307.1218; The enantiomeric purity of the product

determined by HPLC: 74% ee (Chiralpak IB column, *n*-hexane/iPrOH = 90:10, flow rate 1.0 mL/min, λ = 254 nm), t_r (minor) = 47.5 min, t_r (major) = 53.9 min; $[\alpha]^{rt}_D$ = 46.3 (*c* = 0.45 in CH_2Cl_2).

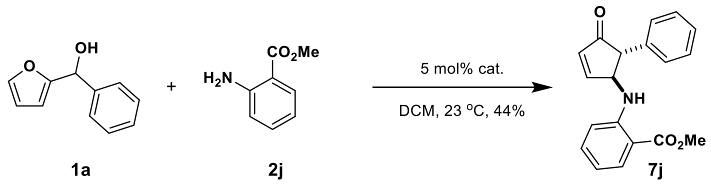

Methyl 2-(((1*S*,5*R*)-4-oxo-5-phenylcyclopent-2-en-1-yl)amino)benzoate (7j): According to the general procedure, the catalyst **8** (4.7 mg, 0.005 mmol, 0.05 equiv) was added to **1a** (20.0 mg, 0.11 mmol, 1.2 equiv) and methyl 2-aminobenzoate (**2j**, 12.4 μL, 0.10 mmol, 1.0 equiv) in 1 mL of anhydrous DCM. The resulting reaction mixture was allowed to stir at room temperature for 5 days before being quenched with 5 mL of saturated aqueous sodium bicarbonate and extracted with DCM (3 × 5 mL). The combined organic layers were dried over $MgSO_4$, filtered, and concentrated in vacuo. The residue was purified via column chromatography to afford cyclopentenone **7j** (12.9 mg, 44%) as a solid. Spectral data matches literature values[3]; The enantiomeric purity of the product determined by HPLC: 80% ee (Chiralpak IB column, *n*-hexane/iPrOH = 90:10, flow rate 1.0 mL/min, λ = 254 nm), t_r (minor) = 14.3 min, t_r (major) = 16.2 min; $[\alpha]^{rt}_D$ = 235.1 (*c* = 0.49 in CH_2Cl_2).

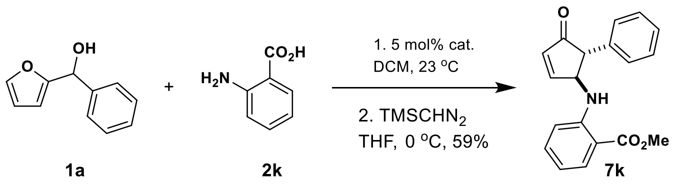

Methyl 2-(((1*S*,5*R*)-4-oxo-5-phenylcyclopent-2-en-1-yl)amino)benzoate (7k): According to the general procedure, the catalyst **8** (4.7 mg, 0.005 mmol, 0.05 equiv) was added to **1a** (20.0 mg, 0.11 mmol, 1.2 equiv) and 2-aminobenzoic acid (**2k**, 13.1 mg, 0.10 mmol, 1.0 equiv) in 1 mL of anhydrous DCM. The resulting reaction mixture was allowed to stir at room temperature for 5 days before being concentrated in vacuo. The residue is dissolved into 0.5 mL of anhydrous THF and cooled to 0 °C. (Trimethysilyl)diazomethane (2 M in hexanes; 0.19 mL, 0.38 mmol, 4.0 equiv) was added dropwise to the reaction and allowed to stir for 15 minutes before being quenched with MeOH. The reaction was then extracted with DCM and water (3 × 10 mL). The combined organic layers were dried over $MgSO_4$, filtered, and concentrated in vacuo. The residue was purified via column chromatography to afford cyclopentenone **7k** (17.3 mg, 59%) as a solid. Spectral data matches literature values[5]; The enantiomeric purity of the product determined by HPLC:

84% ee (Chiralpak IB column, *n*-hexane/iPrOH = 90:10, flow rate 1.0 mL/min, λ = 254 nm), t_r (minor) = 13.9 min, t_r (major) = 15.5 min; $[\alpha]^{rt}_D$ = 254.0 (*c* = 0.42 in CH_2Cl_2).

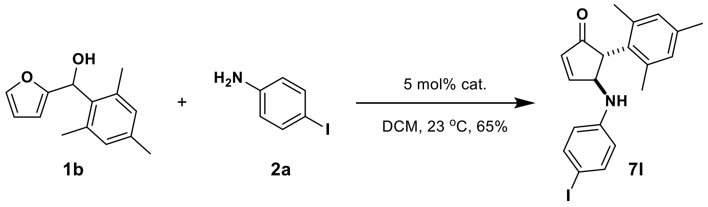

(4*S*,5*R*)-4-((4-Iodophenyl)amino)-5-mesitylcyclopent-2-en-1-one (7l): According to the general procedure, the catalyst **8** (3.8 mg, 0.004 mmol, 0.05 equiv) was added to furan-2-yl(mesityl)methanol (**1b**, 20.0 mg, 0.09 mmol, 1.2 equiv) and **2a** (16.9 mg, 0.08 mmol, 1.0 equiv) in 1 mL of anhydrous DCM. The resulting reaction mixture was allowed to stir at room temperature for 5 days before being quenched with 5 mL of saturated aqueous sodium bicarbonate and extracted with DCM (3 × 5 mL). The combined organic layers were dried over $MgSO_4$, filtered, and concentrated in vacuo. The residue was purified via column chromatography to afford cyclopentenone **7l** (20.9 mg, 65%) as a solid. ^{1}H NMR (500 MHz, Chloroform-*d*) δ 7.62 (dt, *J* = 5.8, 1.6 Hz, 1H), 7.34 – 7.28 (m, 2H), 6.83 (d, *J* = 13.6 Hz, 2H), 6.45 (dt, *J* = 5.9, 1.5 Hz, 1H), 6.27 – 6.20 (m, 2H), 4.75 (dt, *J* = 7.6, 2.4 Hz, 1H), 4.07 (d, *J* = 8.3 Hz, 1H), 3.82 (d, *J* = 3.5 Hz, 1H), 2.25 (s, 3H), 2.11 (s, 3H), 1.99 (s, 3H); ^{13}C NMR (125 MHz, Chloroform-*d*) δ 206.4, 160.0, 146.0, 138.3, 138.0, 137.2, 135.8, 134.9, 131.2, 130.5, 129.5, 115.7, 79.6, 62.2, 56.9, 21.3, 21.0, 20.5, 1.2; IR 3359, 2961, 2919, 2858, 1702, 1587, 1484, 1291, 1248, 1129, 904, 810, 728, 500 cm^{-1}; HRMS (ESI), calculated for $C_{20}H_{20}INO$: (M+Na$^+$) 440.0487; observed 440.0473; The enantiomeric purity of the product determined by HPLC: 17% ee (Chiralpak IB column, *n*-hexane/iPrOH = 90:10, flow rate 1.0 mL/min, λ = 254 nm), t_r (minor) = 15.7 min, t_r (major) = 20.6 min; $[\alpha]^{rt}_D$ = 12.8 (*c* = 0.48 in CH_2Cl_2).

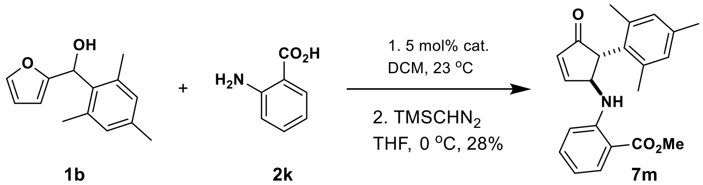

Methyl 2-(((1*S*,5*R*)-5-mesityl-4-oxocyclopent-2-en-1-yl)amino)benzoate (7m): According to the general procedure, the catalyst **8** (3.8 mg, 0.004 mmol, 0.05 equiv) was added to **1b** (20.0 mg, 0.09 mmol, 1.2 equiv) and **2k** (10.6 mg, 0.08 mmol, 1.0 equiv) in 1 mL of anhydrous DCM. The resulting reaction mixture was allowed to stir at room temperature for 5 days before being concentrated in vacuo. The residue is dissolved into 0.5 mL of anhydrous THF and cooled to 0 °C. (Trimethysilyl)diazomethane (2 M in hexanes; 0.15 mL, 0.31 mmol, 4.0 equiv) was added dropwise to the reaction and allowed to stir for 15 minutes before being quenched with MeOH. The reaction was then extracted with DCM and water (3 × 10 mL). The combined organic layers were dried over $MgSO_4$, filtered, and concentrated in vacuo. The residue was purified via column chromatography

to afford cyclopentenone **7m** (7.5 mg, 28%) as a solid. ^{1}H NMR (500 MHz, Chloroform-*d*) δ 8.25 (d, *J* = 7.7 Hz, 1H), 7.91 (dd, *J* = 8.0, 1.7 Hz, 1H), 7.66 (dd, *J* = 5.8, 2.0 Hz, 1H), 7.16 (ddd, *J* = 8.6, 7.1, 1.7 Hz, 1H), 6.85 (s, 1H), 6.81 (s, 1H), 6.61 (ddd, *J* = 8.1, 7.1, 1.0 Hz, 1H), 6.47 (dd, *J* = 5.8, 1.8 Hz, 1H), 6.43 (d, *J* = 8.5 Hz, 1H), 4.91 (ddt, *J* = 7.5, 3.5, 1.8 Hz, 1H), 3.90 (d, *J* = 3.6 Hz, 1H), 3.87 (s, 3H), 2.25 (s, 3H), 2.20 (s, 3H), 2.04 (s, 3H); ^{13}C NMR (125 MHz, Chloroform-*d*) δ 206.4, 169.2, 159.6, 149.8, 138.5, 137.0, 135.8, 134.7, 134.7, 131.9, 131.2, 130.4, 129.4, 116.0, 111.7, 110.8, 60.8, 57.4, 51.8, 21.3, 21.0, 20.9, 20.5; IR 3331, 2951, 2921, 2854, 1705, 1584, 1511, 1316, 1243, 1164, 810, 747, 487 cm^{-1}; HRMS (ESI), calculated for $C_{22}H_{23}NO_3$: (M+Na$^+$) 372.1576; observed 372.1589; The enantiomeric purity of the product determined by HPLC: 28% ee (Chiralpak IB column, *n*-hexane/iPrOH = 90:10, flow rate 1.0 mL/min, λ = 254 nm), t_r (minor) = 15.1 min, t_r (major) = 11.1 min; $[\alpha]^{rt}_D$ = 69.7 (*c* = 0.49 in CH_2Cl_2).

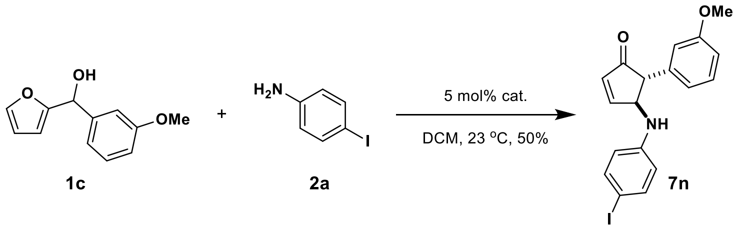

1c 2a 7n

(4*S*,5*R*)-4-((4-Iodophenyl)amino)-5-(3-methoxyphenyl)cyclopent-2-en-1-one (7n): According to the general procedure, the catalyst **8** (4.0 mg, 0.004 mmol, 0.05 equiv) was added to furan-2-yl(3-methoxyphenyl)methanol (**1c**, 20.0 mg, 0.10 mmol, 1.2 equiv) and **2a** (17.9 mg, 0.08 mmol, 1.0 equiv) in 1 mL of anhydrous DCM. The resulting reaction mixture was allowed to stir at room temperature for 5 days before being quenched with 5 mL of saturated aqueous sodium bicarbonate and extracted with DCM (3 × 5 mL). The combined organic layers were dried over $MgSO_4$, filtered, and concentrated in vacuo. The residue was purified via column chromatography to afford cyclopentenone **7n** (16.5 mg, 50%) as a solid. ^{1}H NMR (500 MHz, Chloroform-*d*) δ 7.74 (dd, *J* = 5.7, 2.4 Hz, 1H), 7.40 – 7.35 (m, 2H), 7.29 – 7.23 (m, 1H), 6.84 (ddd, *J* = 8.3, 2.6, 0.9 Hz, 1H), 6.70 (dt, *J* = 7.6, 1.2 Hz, 1H), 6.65 (dd, *J* = 2.5, 1.7 Hz, 1H), 6.42 (dd, *J* = 5.7, 1.7 Hz, 1H), 6.33 – 6.27 (m, 2H), 4.71 (s, 1H), 4.02 (s, 1H), 3.78 (s, 3H), 3.32 (d, *J* = 2.5 Hz, 1H); ^{13}C NMR (125 MHz, Chloroform-*d*) δ 206.0, 161.2, 160.1, 145.9, 139.4, 138.1, 135.3, 130.3, 120.3, 116.1, 114.0, 112.9, 79.8, 63.3, 60.1, 55.4; IR 3367, 2923, 2834, 1703, 1584, 1486, 1315, 1248, 1151, 1038, 806, 755, 696, 497 cm^{-1}; HRMS (ESI), calculated for $C_{18}H_{16}INO_2$: (M+Na$^+$) 428.0124; observed 428.0121; The enantiomeric purity of the product determined by HPLC: 76% ee (Chiralpak IB column, *n*-hexane/iPrOH = 90:10, flow rate 1.0 mL/min, λ = 254 nm), t_r (minor) = 23.4 min, t_r (major) = 33.0 min; $[\alpha]^{rt}_D$ = 37.7 (*c* = 0.51 in CH_2Cl_2).

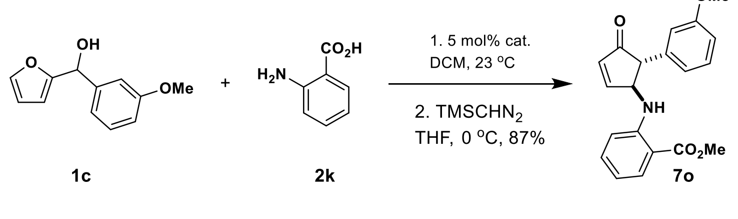

1c 2k 7o

Methyl 2-(((1*S*,5*R*)-5-(3-methoxyphenyl)-4-oxocyclopent-2-en-1-yl)amino)benzoate (7o): According to the general procedure, the catalyst **8** (4.0 mg, 0.004 mmol, 0.05 equiv) was added to **1c** (20.0 mg, 0.10 mmol, 1.2 equiv) and **2k** (11.2 mg, 0.08 mmol, 1.0 equiv) in 1 mL of anhydrous DCM. The resulting reaction mixture was allowed to stir at room temperature for 5 days before being concentrated in vacuo. The residue is dissolved into 0.5 mL of anhydrous THF and cooled to 0 °C. (Trimethysilyl)diazomethane (2 M in hexanes; 0.16 mL, 0.33 mmol, 4.0 equiv) was added dropwise to the reaction and allowed to stir for 15 minutes before being quenched with MeOH. The reaction was then extracted with DCM and water (3 × 10 mL). The combined organic layers were dried over $MgSO_4$, filtered, and concentrated in vacuo. The residue was purified via column chromatography to afford cyclopentenone **7o** (9.9 mg, 36%) as a solid. ^{1}H NMR (500 MHz, Chloroform-*d*) δ 8.18 (d, *J* = 7.6 Hz, 1H), 7.92 (dd, *J* = 8.0, 1.7 Hz, 1H), 7.78 (dd, *J* = 5.7, 2.4 Hz, 1H), 7.30 – 7.24 (m, 1H), 7.22 (ddd, *J* = 8.7, 7.1, 1.8 Hz, 1H), 6.84 (ddd, *J* = 8.3, 2.6, 0.9 Hz, 1H), 6.75 (dt, *J* = 7.6, 1.2 Hz, 1H), 6.70 (t, *J* = 2.1 Hz, 1H), 6.64 (ddd, *J* = 8.1, 7.1, 1.1 Hz, 1H), 6.48 – 6.42 (m, 2H), 4.83 (dq, *J* = 7.4, 2.2 Hz, 1H), 3.87 (s, 3H), 3.78 (s, 3H), 3.39 (d, *J* = 2.6 Hz, 1H); ^{13}C NMR (125 MHz, Chloroform-*d*) δ 206.2, 169.2, 161.2, 160.1, 149.6, 139.6, 135.2, 134.8, 132.0, 130.2, 120.4, 116.1, 114.0, 113.0, 112.4, 111.1, 111.1, 62.3, 60.7, 55.4, 51.8; IR 3333, 3000, 2951, 2837, 1713, 1680, 1581, 1513, 1436, 1318, 1238, 1150, 1046, 747, 699 cm^{-1}; HRMS (ESI), calculated for $C_{20}H_{19}NO_4$: (M+Na$^+$) 360.1212; observed 360.1229; The enantiomeric purity of the product determined by HPLC: 87% ee (Chiralpak IB column, *n*-hexane/iPrOH = 90:10, flow rate 1.0 mL/min, λ = 254 nm), t_r (minor) = 16.3 min, t_r (major) = 20.5 min; $[\alpha]^{rt}_D$ = 217.8 (*c* = 0.53 in CH_2Cl_2).

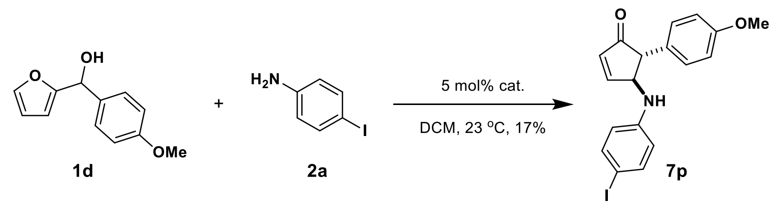

(4*S*,5*R*)-4-((4-Iodophenyl)amino)-5-(4-methoxyphenyl)cyclopent-2-en-1-one (7p): According to the general procedure, the catalyst **8** (4.0 mg, 0.004 mmol, 0.05 equiv) was added to furan-2-yl(4-methoxyphenyl)methanol (**1d**, 20.0 mg, 0.10 mmol, 1.2 equiv) and **2a** (17.9 mg, 0.08 mmol, 1.0 equiv) in 1 mL of anhydrous DCM. The resulting reaction mixture was allowed to stir at room temperature for 5 days before being quenched with 5 mL of saturated aqueous sodium bicarbonate and extracted with DCM (3 × 5 mL). The combined organic layers were dried over $MgSO_4$, filtered, and concentrated in vacuo. The residue was purified via column chromatography to afford cyclopentenone **7p** (5.6 mg, 17%) as a solid. Spectral data matches literature values[3]; The enantiomeric purity of the product determined by HPLC: 75% ee (Chiralpak IB column, *n*-hexane/iPrOH = 90:10, flow rate 1.0 mL/min, λ = 254 nm), t_r (minor) = 27.1 min, t_r (major) = 38.0 min; $[\alpha]^{rt}_D$ = 26.6 (*c* = 0.51 in CH_2Cl_2).

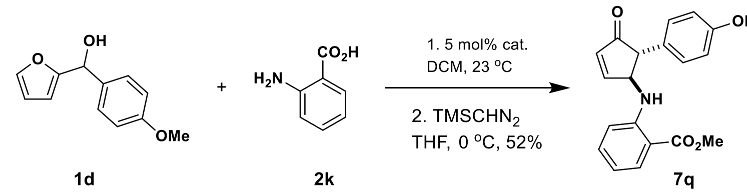

Methyl 2-(((1*S*,5*R*)-5-(4-methoxyphenyl)-4-oxocyclopent-2-en-1-yl)amino)benzoate (7q): According to the general procedure, the catalyst **8** (4.0 mg, 0.004 mmol, 0.05 equiv) was added to **1d** (20.0 mg, 0.10 mmol, 1.2 equiv) and **2k** (11.2 mg, 0.08 mmol, 1.0 equiv) in 1 mL of anhydrous DCM. The resulting reaction mixture was allowed to stir at room temperature for 5 days before being concentrated in vacuo. The residue is dissolved into 0.5 mL of anhydrous THF and cooled to 0 °C. (Trimethysilyl)diazomethane (2 M in hexanes; 0.16 mL, 0.33 mmol, 4.0 equiv) was added dropwise to the reaction and allowed to stir for 15 minutes before being quenched with MeOH. The reaction was then extracted with DCM and water (3 × 10 mL). The combined organic layers were dried over $MgSO_4$, filtered, and concentrated in vacuo. The residue was purified via column chromatography to afford cyclopentenone **7q** (14.3 mg, 52%) as a solid. ^{1}H NMR (500 MHz, Chloroform-*d*) δ 8.18 (d, *J* = 7.5 Hz, 1H), 7.92 (dt, *J* = 8.0, 1.4 Hz, 1H), 7.76 (ddd, *J* = 5.9, 2.4, 1.0 Hz, 1H), 7.22 (ddd, *J* = 8.5, 7.1, 1.6 Hz, 1H), 7.11 – 7.06 (m, 2H), 6.91 – 6.86 (m, 2H), 6.63 (ddt, *J* = 8.2, 7.1, 1.1 Hz, 1H), 6.47 – 6.41 (m, 2H), 4.81 – 4.75 (m, 1H), 3.86 (s, 3H), 3.80 (s, 3H), 3.38 (d, *J* = 2.6 Hz, 1H); ^{13}C NMR (125 MHz, Chloroform-*d*) δ 169.2, 161.0, 159.1, 149.7, 135.1, 134.8, 132.0, 130.1, 129.2, 116.1, 114.7, 112.4, 111.0, 62.4, 60.0, 55.5, 51.8; IR 3331, 2996, 2950, 2836, 1716, 1677, 1582, 1511, 1436, 1239, 1178, 1032, 746, 524 cm^{-1}; HRMS (ESI), calculated for $C_{20}H_{19}NO_4$: (M+Na$^+$) 360.1212; observed 360.1215; The enantiomeric purity of the product determined by HPLC: 86% ee (Chiralpak IB column, *n*-hexane/iPrOH = 90:10, flow rate 1.0 mL/min, λ = 254 nm), t_r (minor) = 18.0 min, t_r (major) = 22.7 min; $[\alpha]^{rt}_D$ = 300.3 (*c* = 0.38 in CH_2Cl_2).

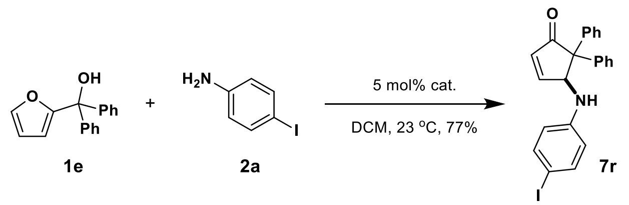

(*S*)-4-((4-Iodophenyl)amino)-5,5-diphenylcyclopent-2-en-1-one (7r): According to the general procedure, the catalyst **8** (3.3 mg, 0.003 mmol, 0.05 equiv) was added to furan-2-yldiphenylmethanol (**1e**, 20.0 mg, 0.08 mmol, 1.2 equiv) and **2a** (14.6 mg, 0.07 mmol, 1.0 equiv) in 1 mL of anhydrous DCM. The resulting reaction mixture was allowed to stir at room temperature for 5 days before being quenched with 5 mL of saturated aqueous sodium bicarbonate and extracted with DCM (3 × 5 mL). The combined organic layers were dried over $MgSO_4$, filtered, and concentrated in vacuo. The residue was purified via column chromatography to afford cyclohentenone **7r** (23.1 mg, 77%) as a solid. ^{1}H NMR (500 MHz, Chloroform-*d*) δ 7.73 (dd, *J* = 5.9, 2.3 Hz, 1H), 7.46 (dd, *J* = 7.5, 1.9 Hz, 2H), 7.37 – 7.22 (m, 8H), 7.00 – 6.95 (m, 2H), 6.47 (dd, *J* = 5.8, 1.8 Hz, 1H), 6.22 (d, *J* = 8.6 Hz, 2H), 5.49 (dt, *J* = 10.0, 2.2 Hz, 1H), 3.35 (d, *J* = 9.9 Hz, 1H); ^{13}C NMR (125 MHz, Chloroform-*d*) δ 206.8, 161.3, 145.7, 140.7, 140.3, 138.1, 134.2, 129.9, 128.7, 128.4,

128.4, 127.7, 127.6, 115.7, 79.2, 65.1, 63.5; IR 3381, 3024, 2920, 1697, 1587, 1482, 1315, 1157, 805, 742, 701, 507 cm^{-1}; HRMS (ESI), calculated for $C_{23}H_{18}INO$: $(M+Na^+)$ 474.0331; observed 474.0347; The enantiomeric purity of the product determined by HPLC: 41% ee (Chiralpak IB column, *n*-hexane/iPrOH = 90:10, flow rate 1.0 mL/min, λ = 254 nm), t_r (minor) = 15.0 min, t_r (major) = 20.9 min; $[\alpha]^{rt}_D$ = 136.9 (*c* = 0.46 in CH_2Cl_2).

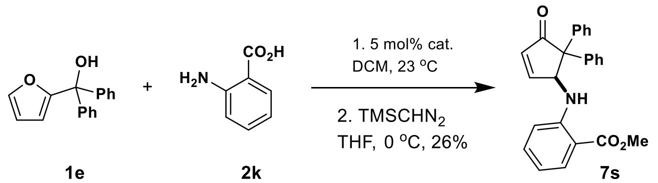

1e 2k 7s

Methyl (*S*)-2-((4-oxo-5,5-diphenylcyclopent-2-en-1-yl)amino)benzoate (7s): According to the general procedure, the catalyst **8** (3.3 mg, 0.003 mmol, 0.05 equiv) was added to (**1e**, 20.0 mg, 0.08 mmol, 1.2 equiv) and **2k** (9.1 mg, 0.07 mmol, 1.0 equiv) in 1 mL of anhydrous DCM. The resulting reaction mixture was allowed to stir at room temperature for 5 days before being concentrated in vacuo. The residue is dissolved into 0.5 mL of anhydrous THF and cooled to 0 °C. (Trimethysilyl)diazomethane (2 M in hexanes; 0.13 mL, 0.27 mmol, 4.0 equiv) was added dropwise to the reaction and allowed to stir for 15 minutes before being quenched with MeOH. The reaction was then extracted with DCM and water (3 × 10 mL). The combined organic layers were dried over $MgSO_4$, filtered, and concentrated in vacuo. The residue was purified via column chromatography to afford cyclopentenone **7s** (6.6 mg, 26%) as a solid. ^1H NMR (500 MHz, Chloroform-*d*) δ 7.78 (dd, *J* = 5.8, 2.5 Hz, 1H), 7.75 (dd, *J* = 8.0, 1.7 Hz, 1H), 7.49 – 7.45 (m, 3H), 7.37 – 7.27 (m, 4H), 7.16 – 7.11 (m, 3H), 7.00 – 6.96 (m, 2H), 6.75 (d, *J* = 8.5 Hz, 1H), 6.58 (ddd, *J* = 8.1, 7.1, 1.0 Hz, 1H), 6.47 (dd, *J* = 5.8, 1.8 Hz, 1H), 5.70 (dt, *J* = 9.6, 2.2 Hz, 1H), 3.66 (s, 3H); ^{13}C NMR (125 MHz, Chloroform-*d*) δ 207.1, 168.3, 161.1, 149.1, 141.0, 140.1, 134.5, 134.1, 132.0, 130.2, 128.7, 128.3, 128.0, 127.5, 127.2, 115.8, 112.0, 111.5, 65.6, 63.3, 51.6; IR 3313, 2923, 2853, 1691, 1577, 1495, 1439, 1259, 1236, 745, 698 cm^{-1}; HRMS (ESI), calculated for $C_{25}H_{21}NO_3$: $(M+Na^+)$ 406.1419; observed 406.1405; The enantiomeric purity of the product determined by HPLC: 72% ee (Chiralpak IB column, *n*-hexane/iPrOH = 90/10, flow rate 1.0 mL/min, λ = 254 nm), t_r (minor) = 11.7 min, t_r (major) = 14.7 min; $[\alpha]^{rt}_D$ = 161.0 (*c* = 0.50 in CH_2Cl_2).

References

[1] Csakÿ, A. G.; Mba, M.; Plumet, J. *Synlett* **2003**, *13*, 2092-2094.

[2] Gheewala, C. D.; Collins, B. E.; Lambert, T. H. *Science* **2016**, *351*, 961-965.

[3] Veits, G. K.; Wenz, D. R.; Read de Alaniz, J. *Angew. Chem Int. Ed.* **2010**, *49*, 9484.

[4] Leboeuf, D.; Schulz, E.; Gandon, V. *Org. Lett.* **2014**, *16*, 6464.

[5] Cai, Y.; Tang, Y.; Atodiresei, I.; Rueping, M. *Angew. Chem. Int. Ed.* **2016**, *55,* 14126.

NMR Spectra

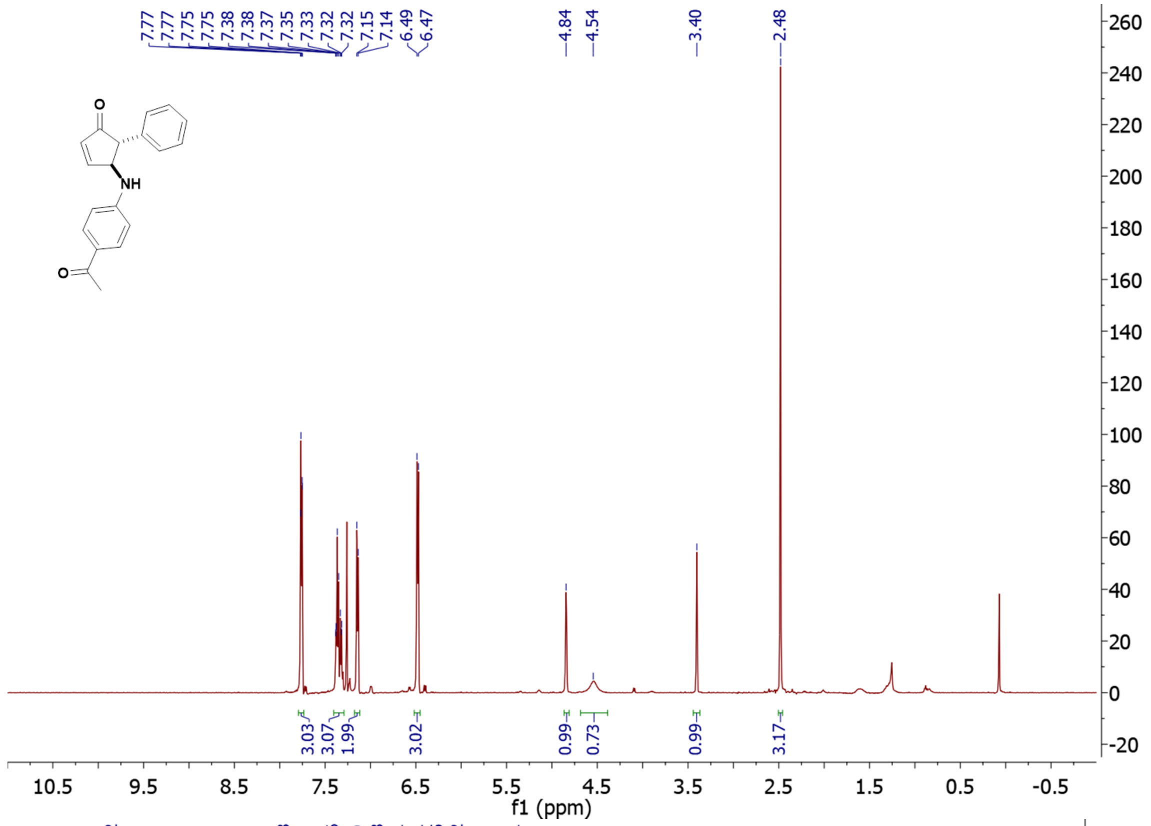
7.77 7.77 7.75 7.75 7.38 7.38 7.37 7.35 7.33 7.32 7.32 7.15 7.14 6.49 6.47
4.84
4.54
3.40
2.48
NH
O
3.03 3.07 1.99 3.02 0.99 0.73 0.99 3.17
f1 (ppm)

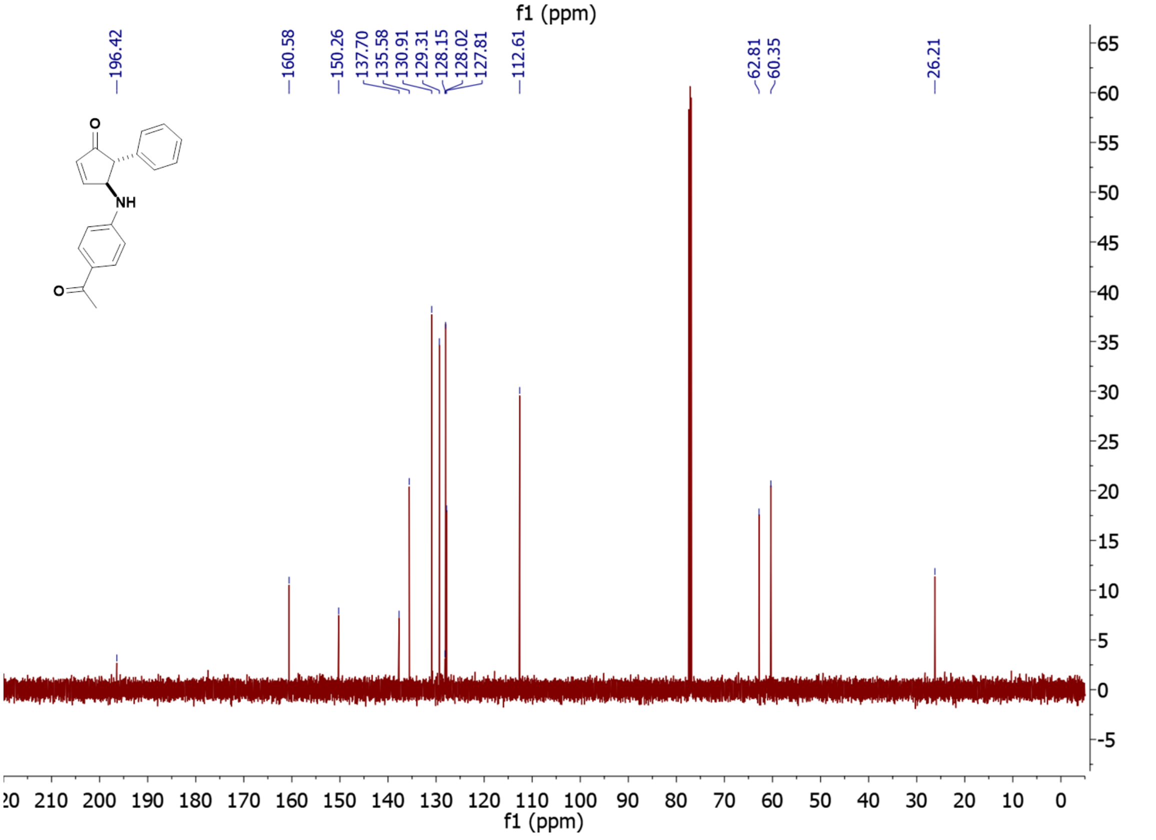
196.42
160.58
150.26
137.70 135.58 130.91 129.31 128.15 128.02 127.81
112.61
62.81 60.35
26.21
NH
O
f1 (ppm)

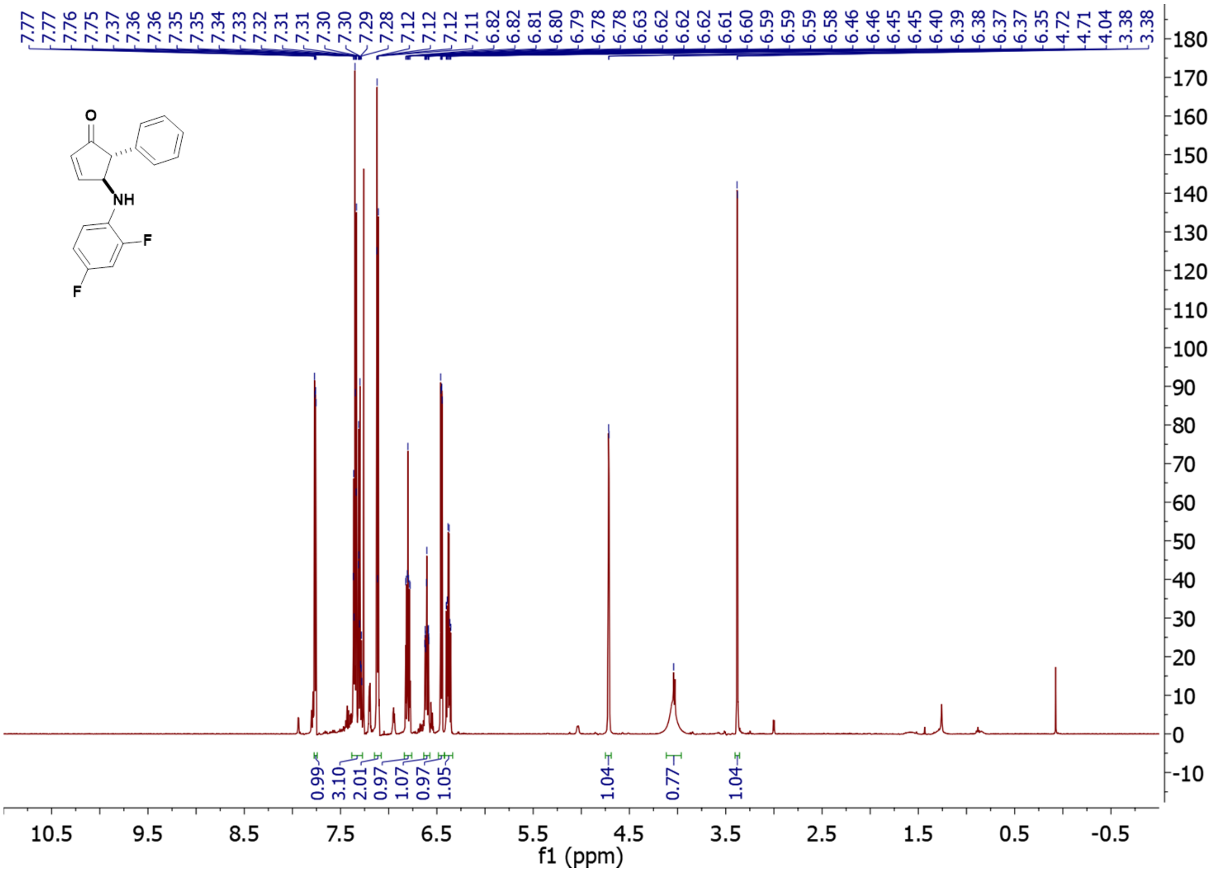

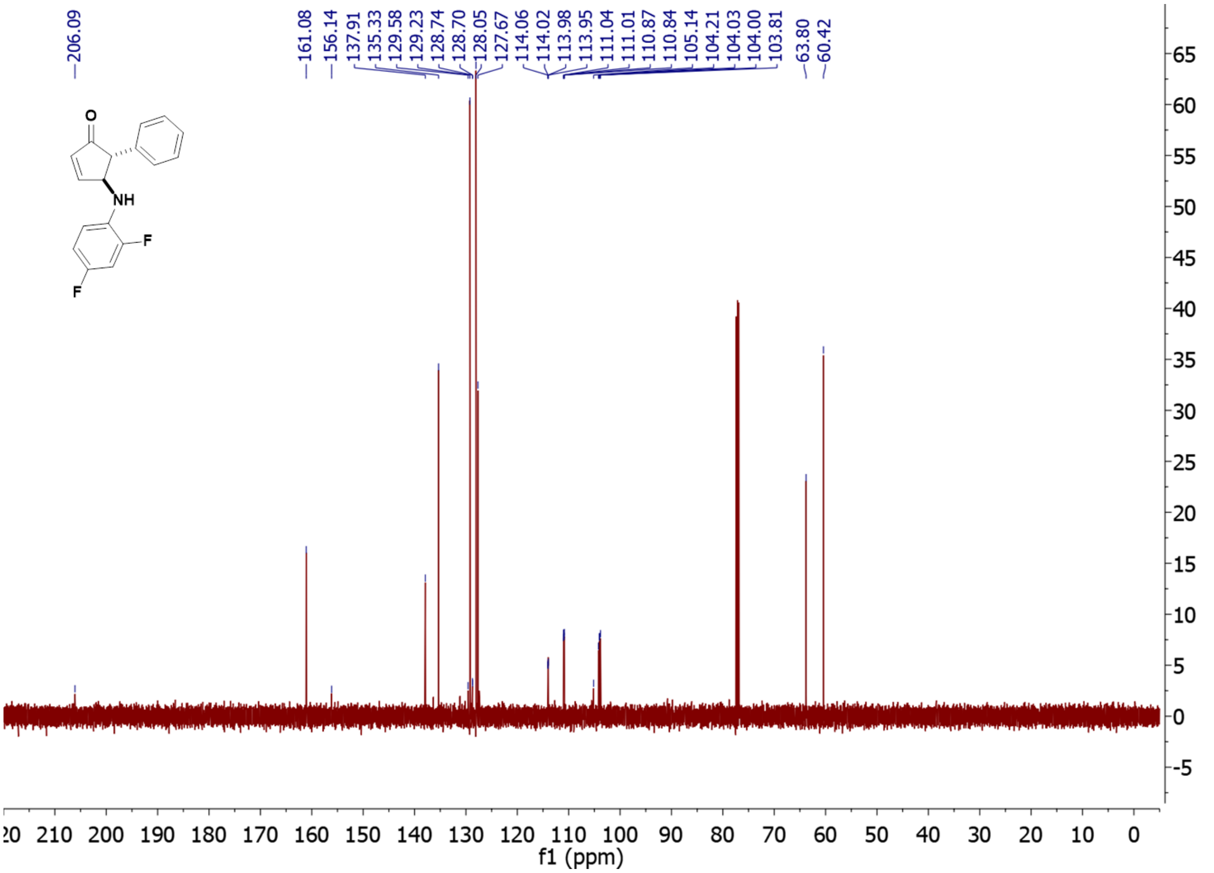

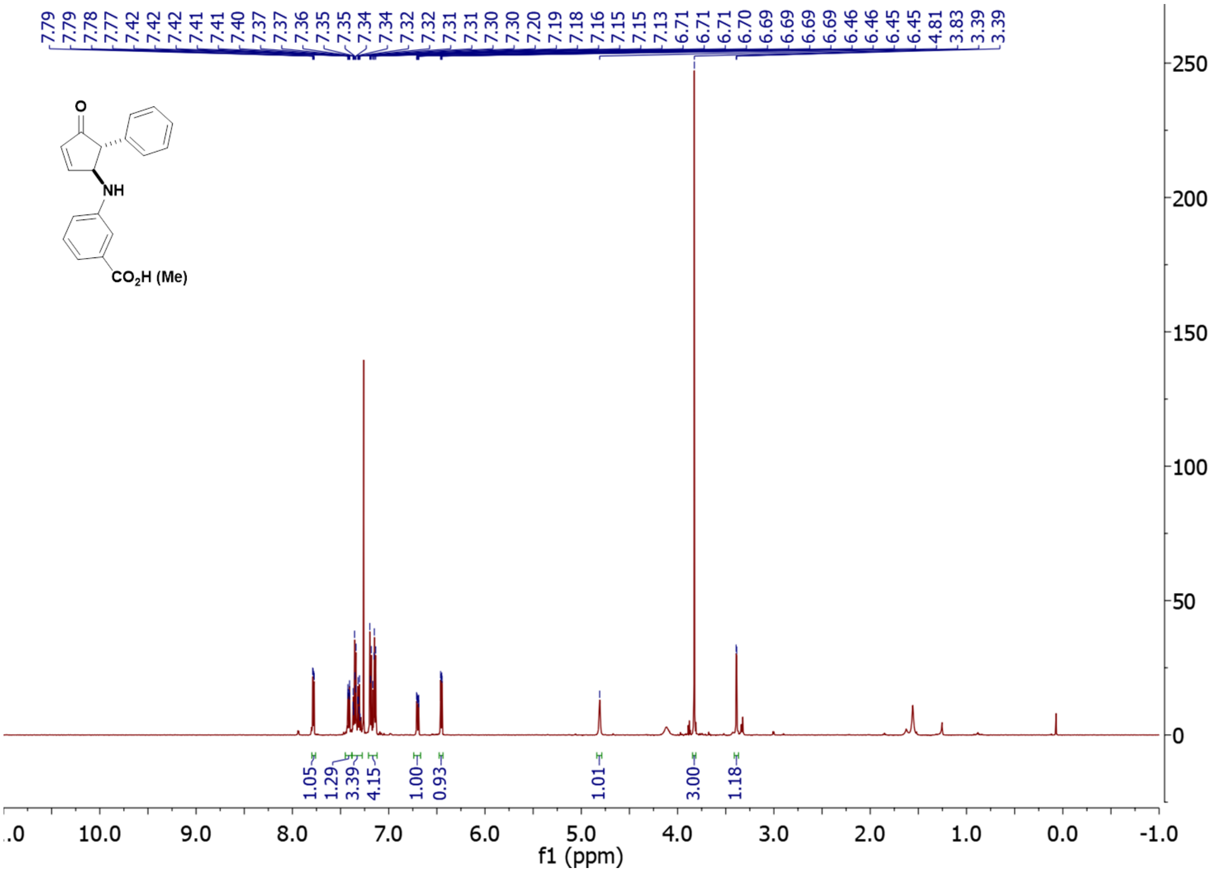
7.79
7.79
7.78
7.77
7.42
7.42
7.42
7.41
7.41
7.40
7.37
7.37
7.36
7.35
7.35
7.34
7.34
7.32
7.32
7.31
7.31
7.30
7.30
7.20
7.19
7.18
7.16
7.15
7.15
7.13
6.71
6.71
6.70
6.69
6.69
6.69
6.69
6.46
6.46
6.45
6.45
4.81
3.83
3.39
3.39
O
NH
CO2H (Me)
1.05
1.29
3.39
4.15
1.00
0.93
1.01
3.00
1.18
f1 (ppm)

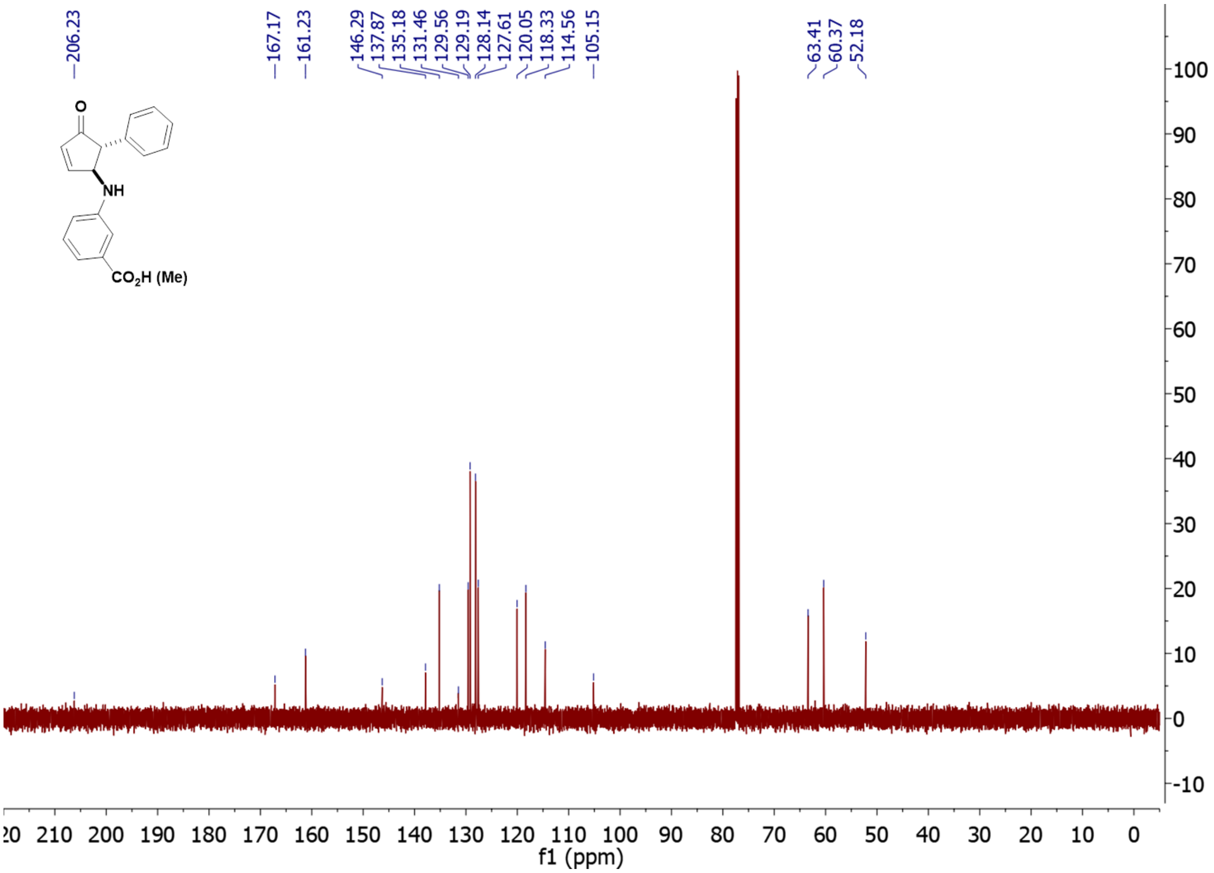
206.23
167.17
161.23
146.29
137.87
135.18
131.46
129.56
129.19
128.14
127.61
120.05
118.33
114.56
105.15
63.41
60.37
52.18
O
NH
CO2H (Me)
f1 (ppm)

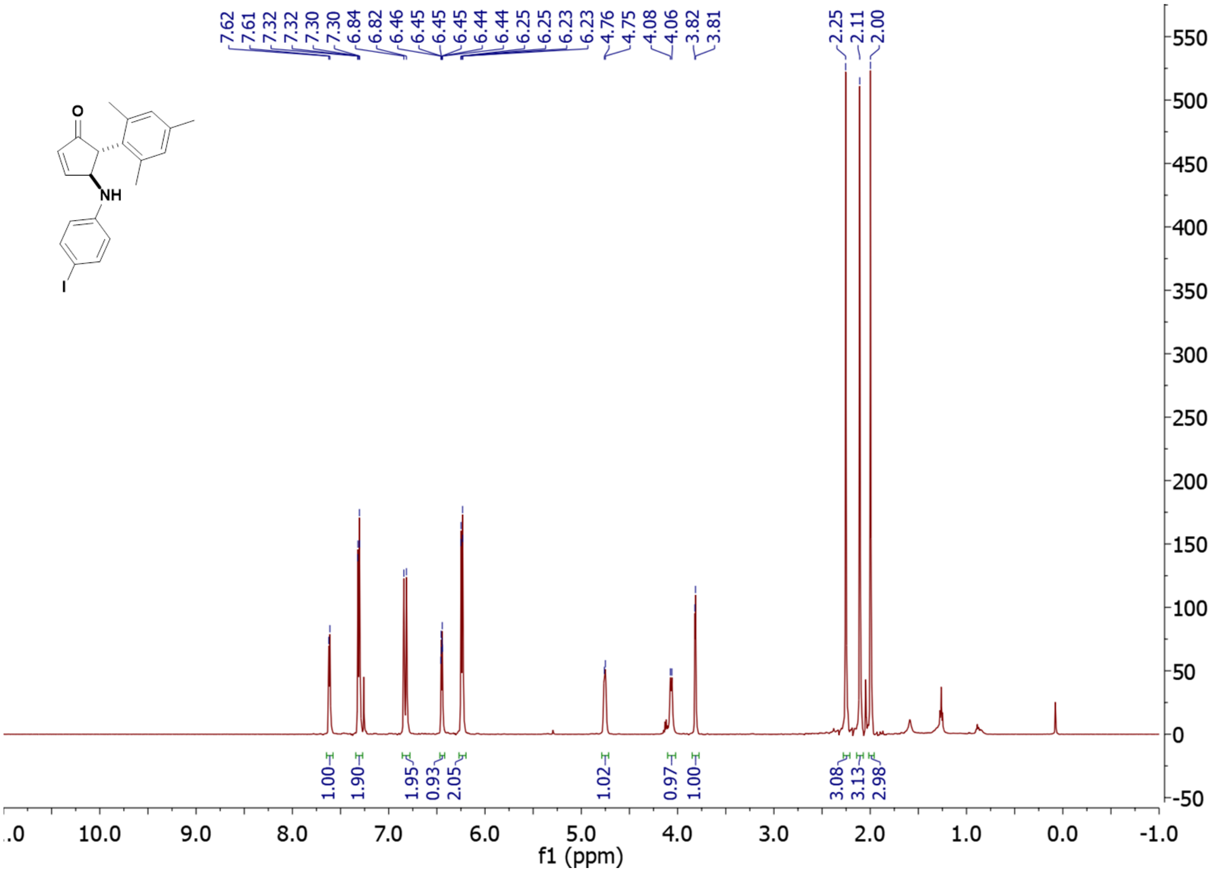
7.62
7.61
7.32
7.32
7.30
7.30
6.84
6.82
6.46
6.45
6.45
6.45
6.44
6.44
6.25
6.25
6.23
6.23
4.76
4.75
4.08
4.06
3.82
3.81
2.25
2.11
2.00
NH
O
I
1.00
1.90
1.95
0.93
2.05
1.02
0.97
1.00
3.08
3.13
2.98
f1 (ppm)

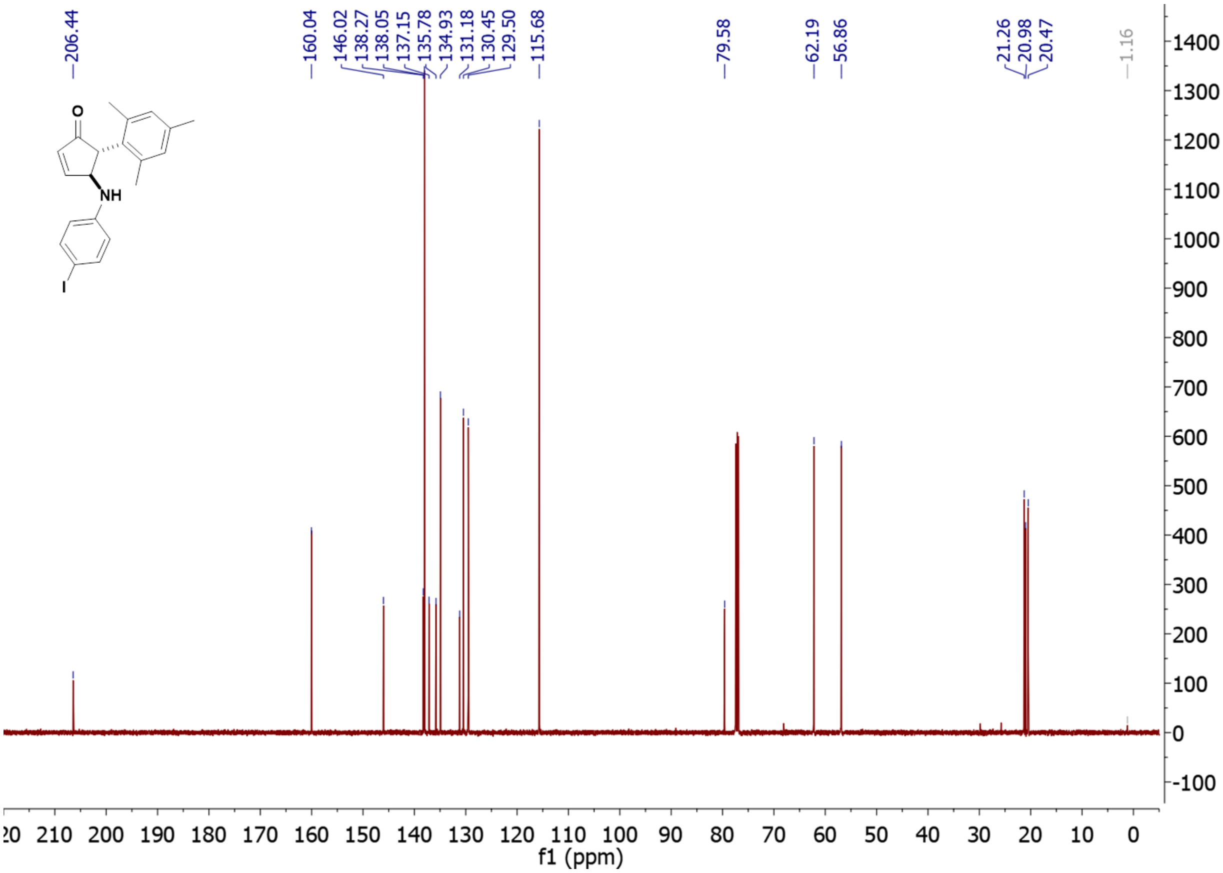
206.44
160.04
146.02
138.27
138.05
137.15
135.78
134.93
131.18
130.45
129.50
115.68
79.58
62.19
56.86
21.26
20.98
20.47
1.16
NH
O
I
f1 (ppm)

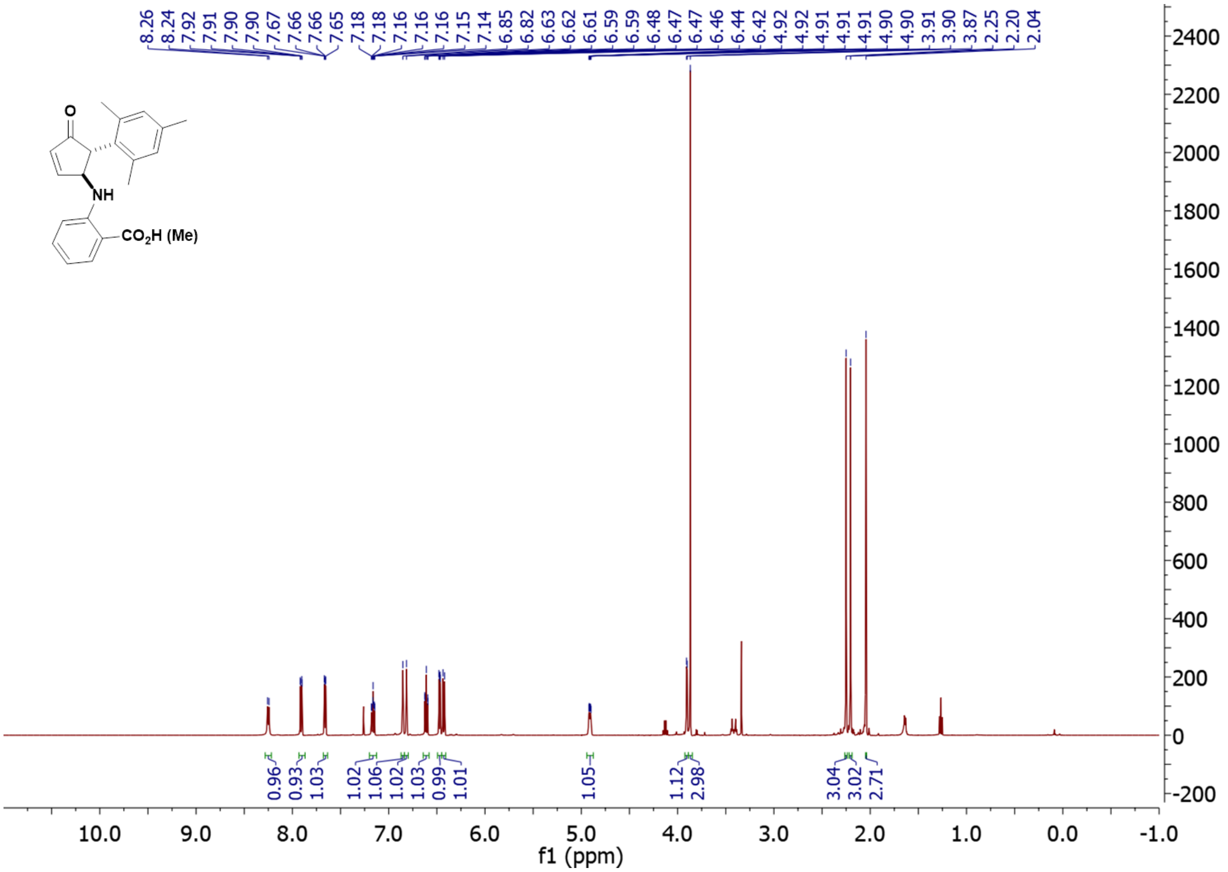
8.26 8.24 7.92 7.91 7.90 7.90 7.67 7.66 7.66 7.65 7.18 7.18 7.16 7.16 7.16 7.15 7.14 6.85 6.82 6.63 6.62 6.61 6.59 6.59 6.48 6.47 6.47 6.46 6.44 6.42 4.92 4.92 4.91 4.91 4.91 4.90 3.91 3.90 3.87 2.25 2.20 2.04
O
NH
CO_2H (Me)
0.96 0.93 1.03 1.02 1.06 1.02 1.03 0.99 1.01 1.05 1.12 2.98 3.04 3.02 2.71
f1 (ppm)

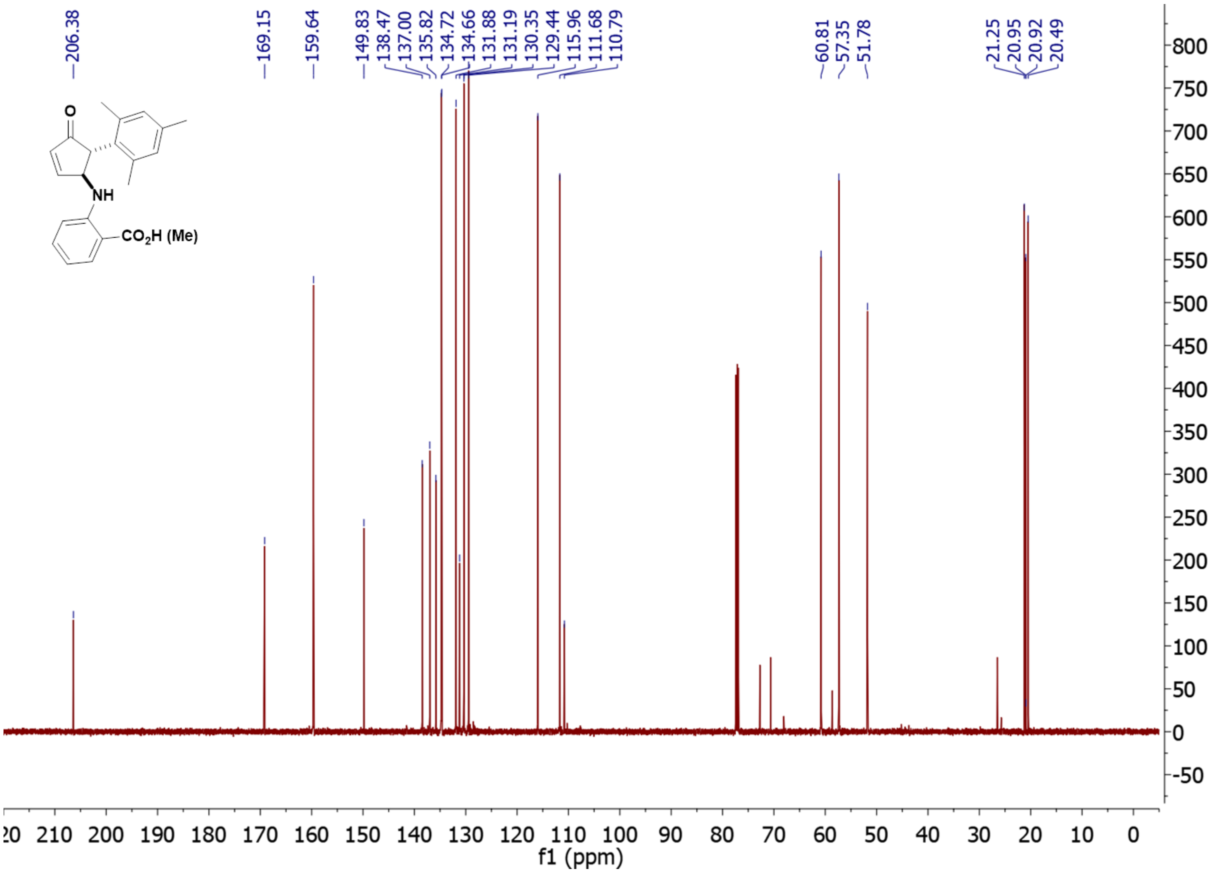
206.38 169.15 159.64 149.83 138.47 137.00 135.82 134.72 134.66 131.88 131.19 130.35 129.44 115.96 111.68 110.79 60.81 57.35 51.78 21.25 20.95 20.92 20.49
O
NH
CO_2H (Me)
f1 (ppm)

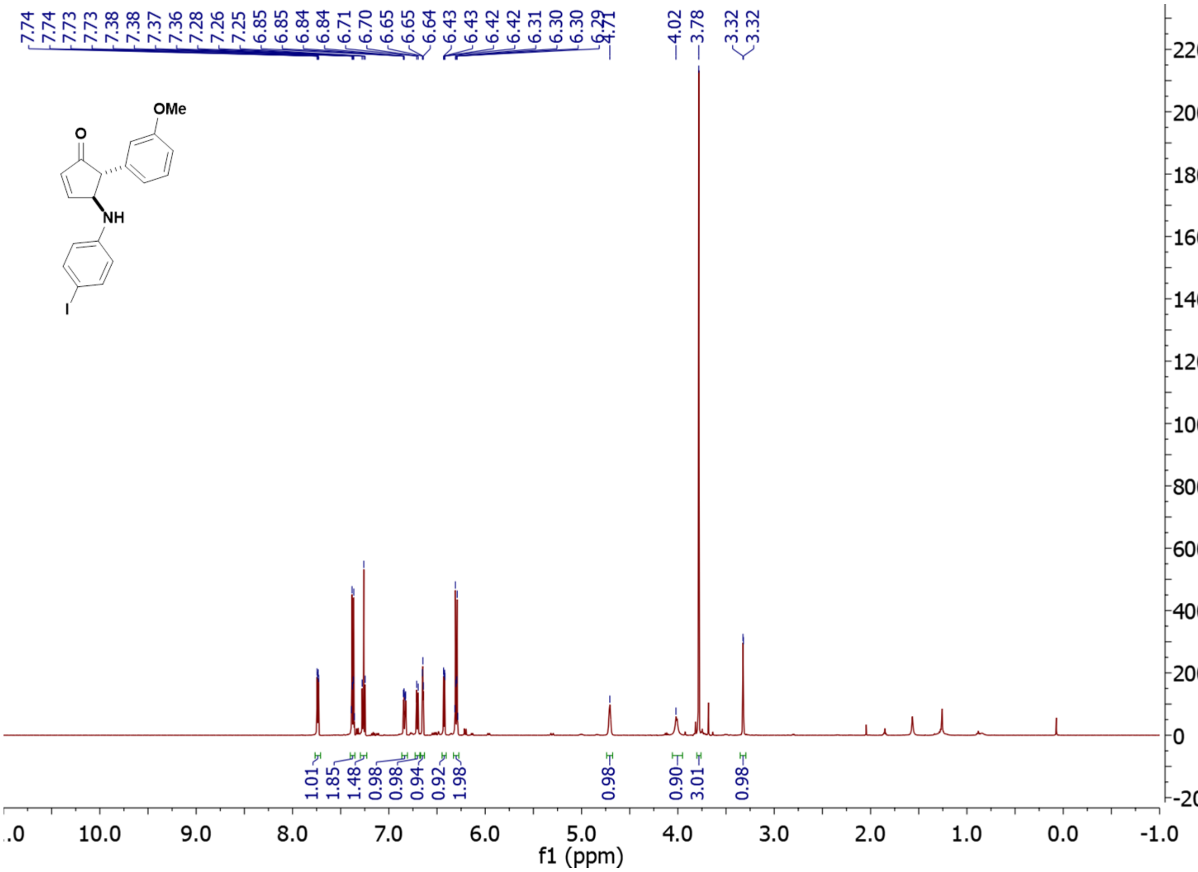

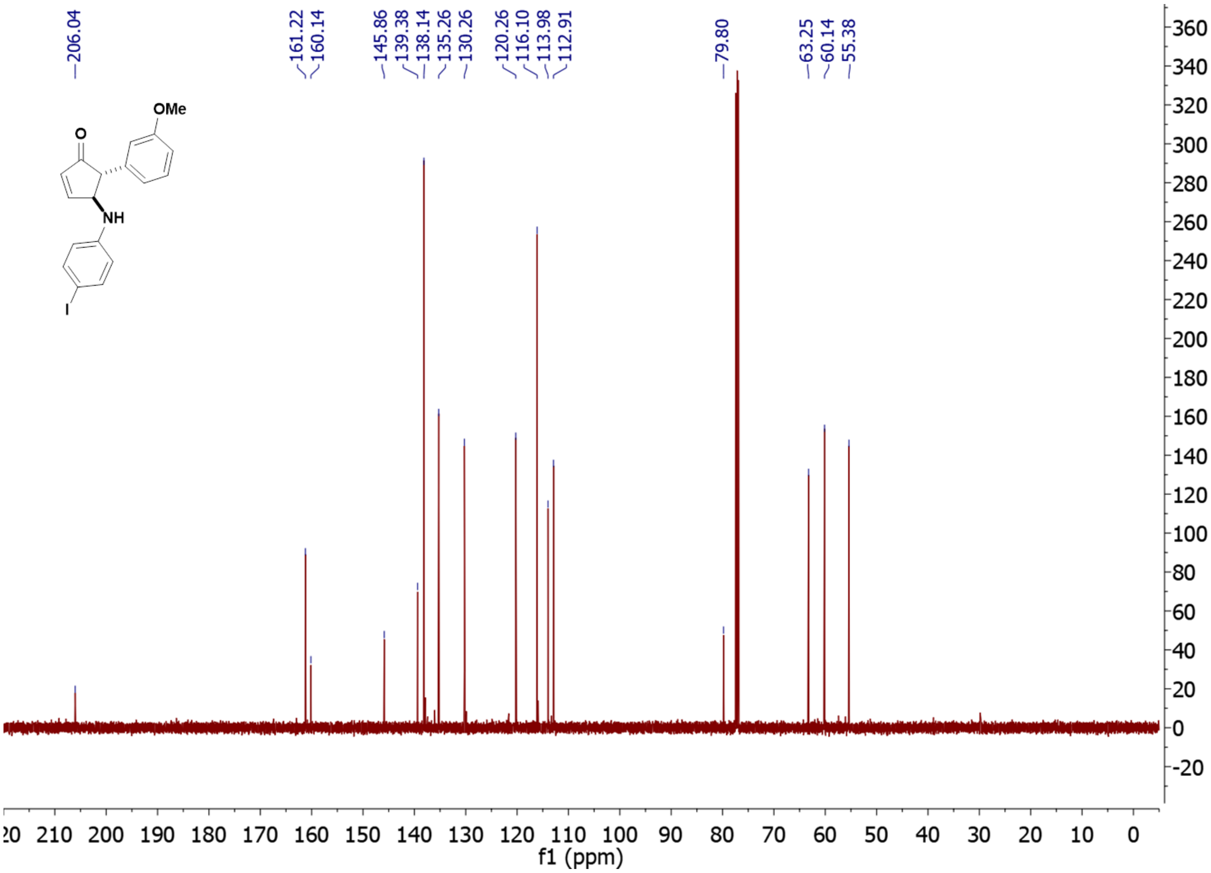

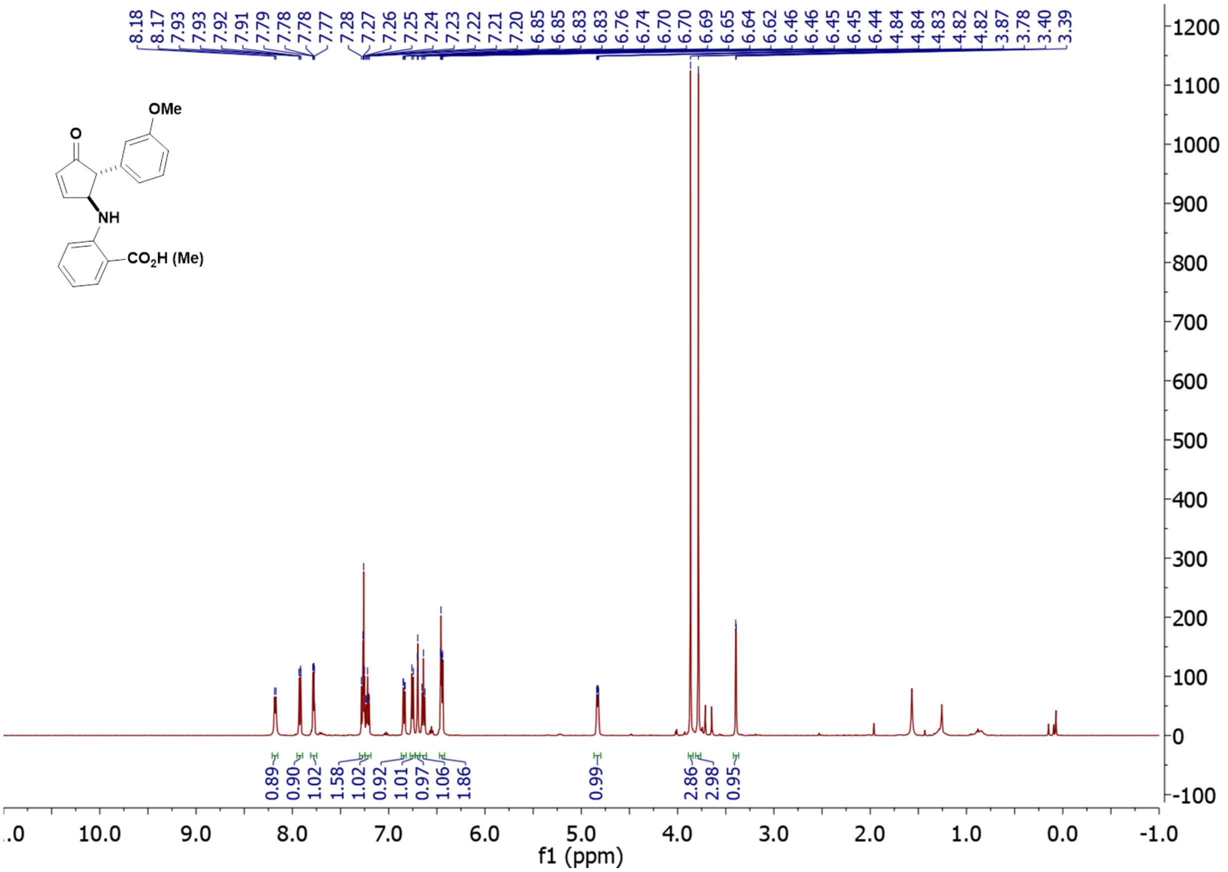
8.18 8.17 7.93 7.93 7.92 7.91 7.79 7.78 7.78 7.77 7.28 7.27 7.26 7.25 7.24 7.23 7.22 7.21 7.20 6.85 6.85 6.83 6.83 6.76 6.74 6.70 6.70 6.69 6.65 6.64 6.62 6.46 6.46 6.45 6.45 6.44 4.84 4.83 4.82 4.82 3.87 3.78 3.40 3.39
OMe
O
NH
CO_2H (Me)
0.89 0.90 1.02 1.58 1.02 0.92 1.01 0.97 1.06 1.86 0.99 2.86 2.98 0.95
f1 (ppm)

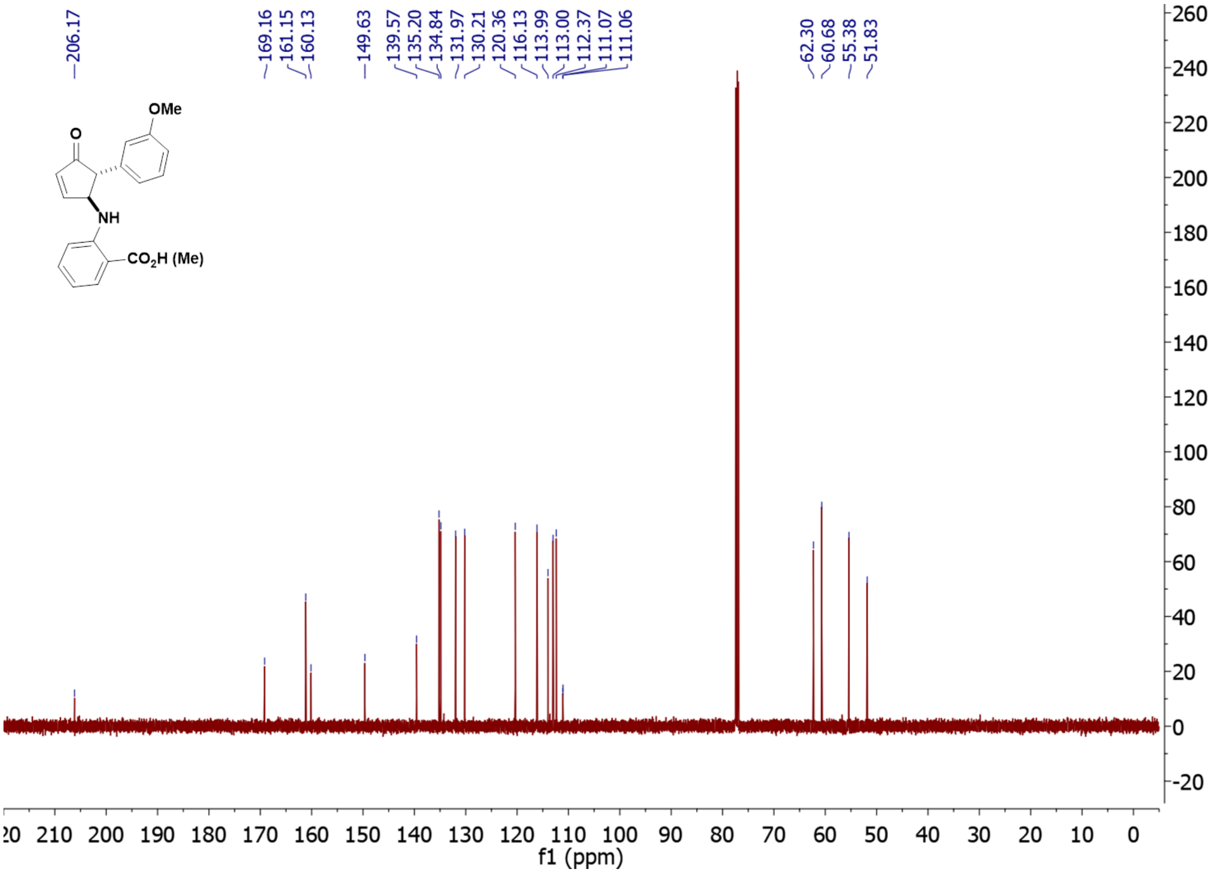
206.17
169.16 161.15 160.13 149.63 139.57 135.20 134.84 131.97 130.21 120.36 116.13 113.99 113.00 112.37 111.07 111.06
62.30 60.68 55.38 51.83
OMe
O
NH
CO_2H (Me)
f1 (ppm)

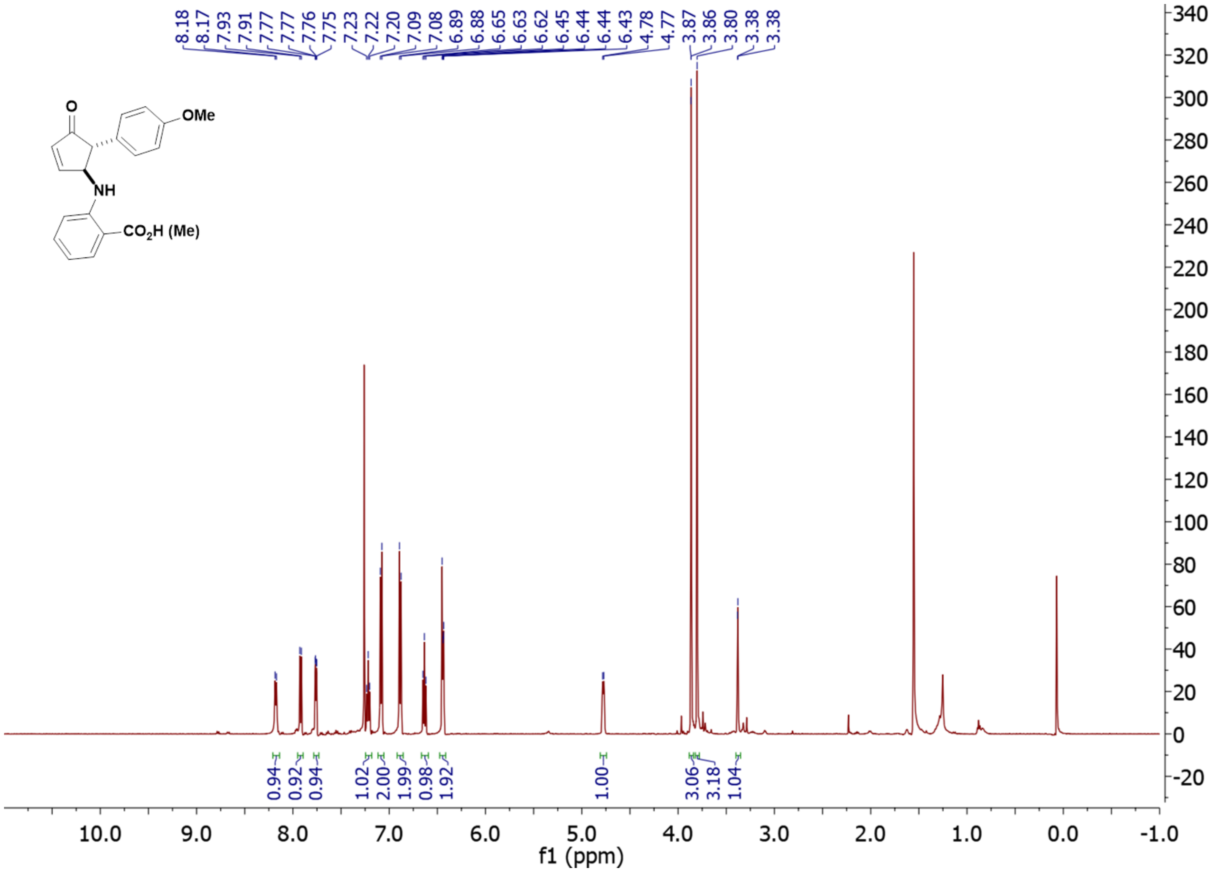
8.18
8.17
7.93
7.91
7.77
7.77
7.76
7.75
7.23
7.22
7.20
7.09
7.08
6.89
6.88
6.65
6.63
6.62
6.45
6.44
6.44
6.43
4.78
4.77
3.87
3.86
3.80
3.38
3.38
O
OMe
NH
CO_2H (Me)
0.94
0.92
0.94
1.02
2.00
1.99
0.98
1.92
1.00
3.06
3.18
1.04
f1 (ppm)

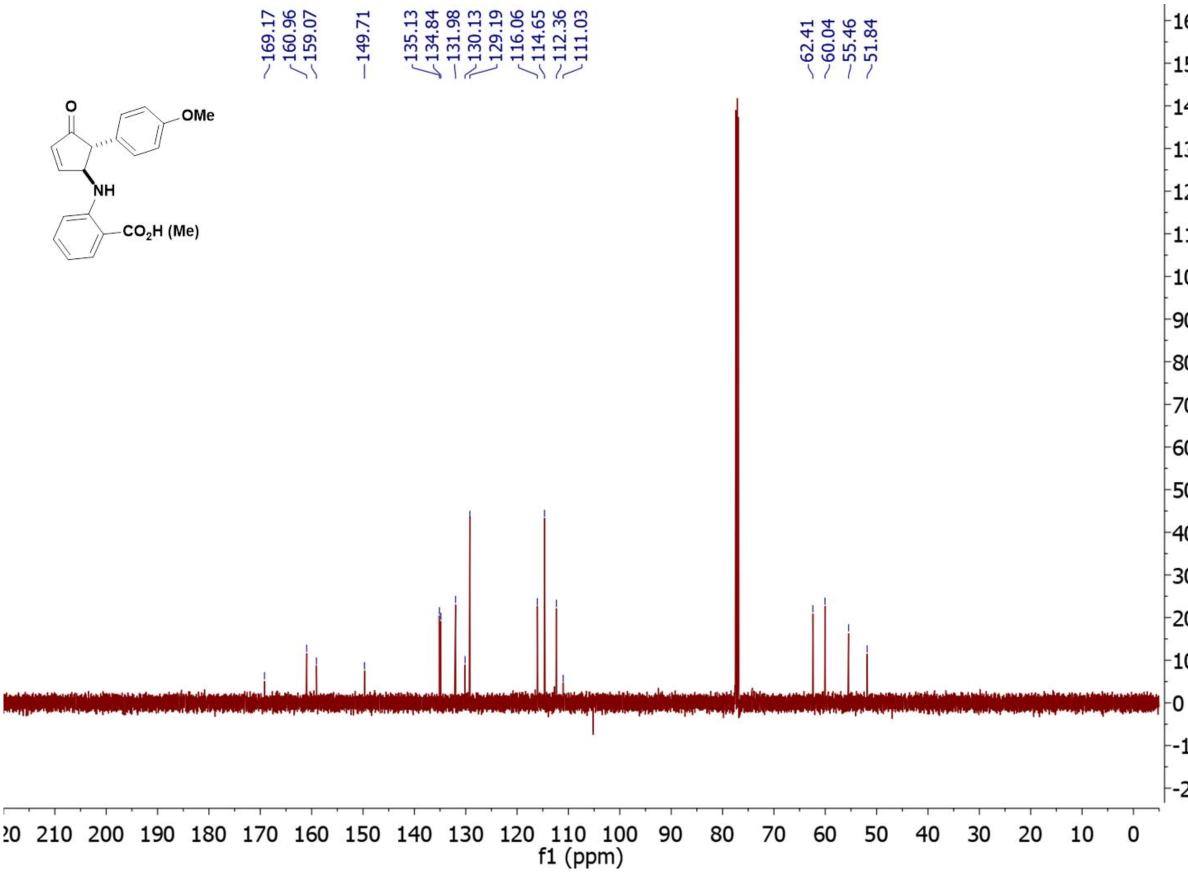
169.17
160.96
159.07
149.71
135.13
134.84
131.98
130.13
129.19
116.06
114.65
112.36
111.03
62.41
60.04
55.46
51.84
O
OMe
NH
CO_2H (Me)
f1 (ppm)

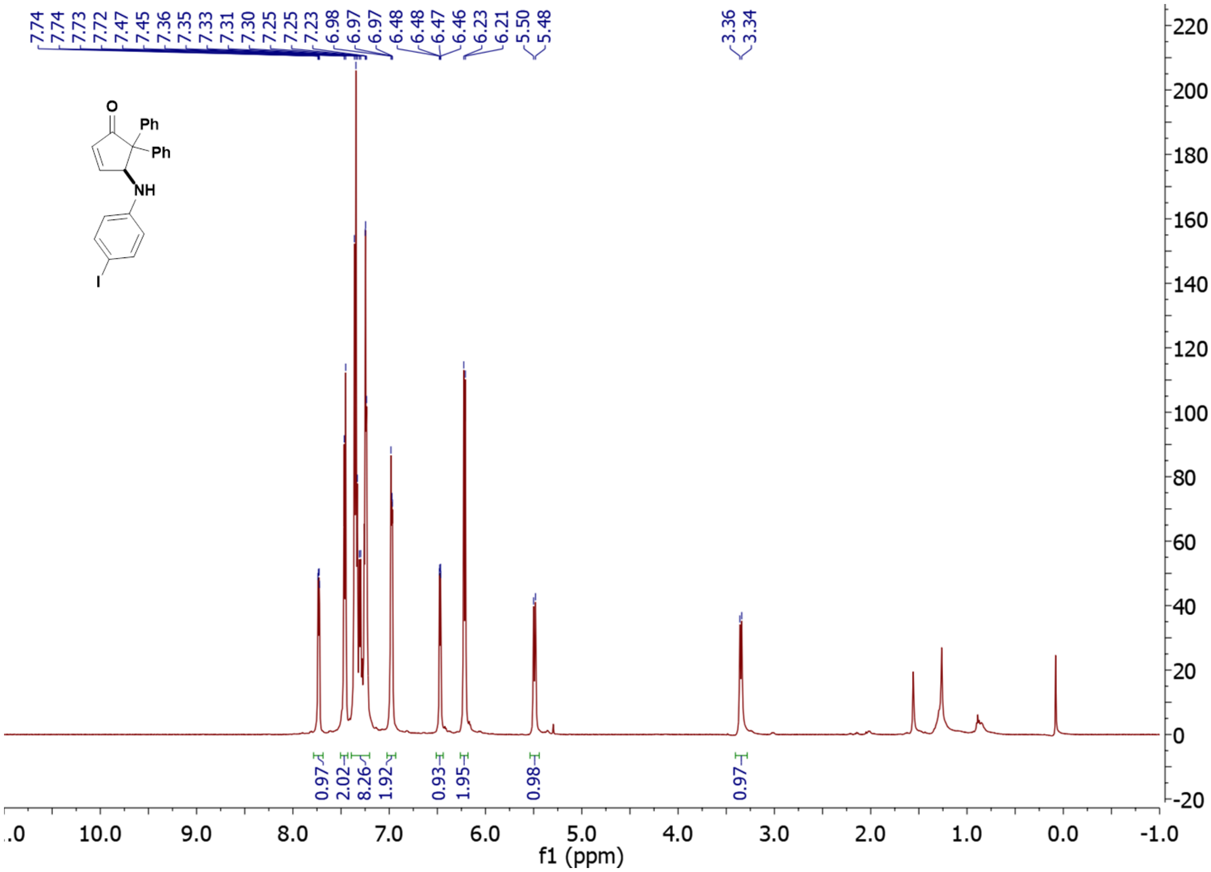
7.74
7.74
7.73
7.72
7.47
7.45
7.36
7.35
7.33
7.31
7.30
7.25
7.25
7.23
6.98
6.97
6.97
6.48
6.48
6.47
6.46
6.23
6.21
5.50
5.48
3.36
3.34
O
Ph
Ph
NH
I
0.97
2.02
8.26
1.92
0.93
1.95
0.98
0.97
f1 (ppm)

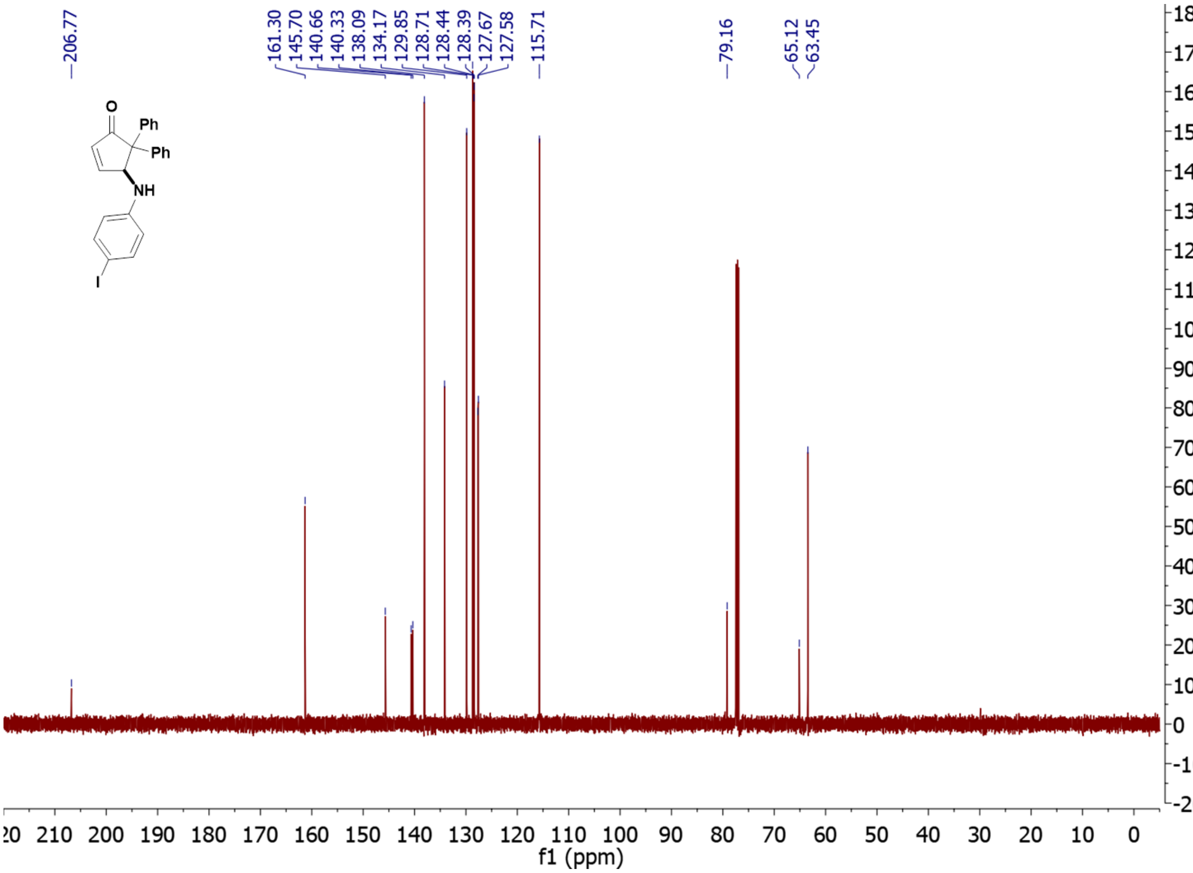
206.77
161.30
145.70
140.66
140.33
138.09
134.17
129.85
128.71
128.44
128.39
127.67
127.58
115.71
79.16
65.12
63.45
O
Ph
Ph
NH
I
f1 (ppm)

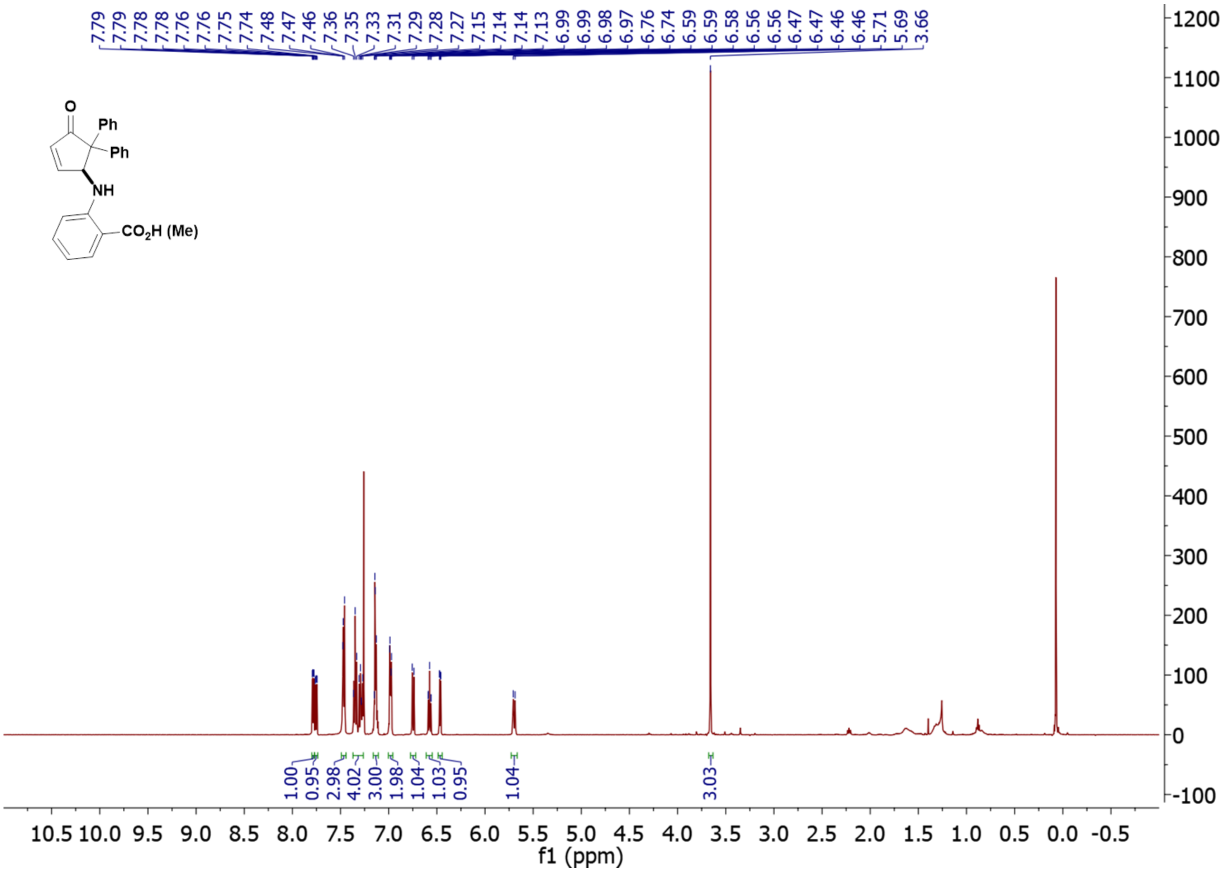

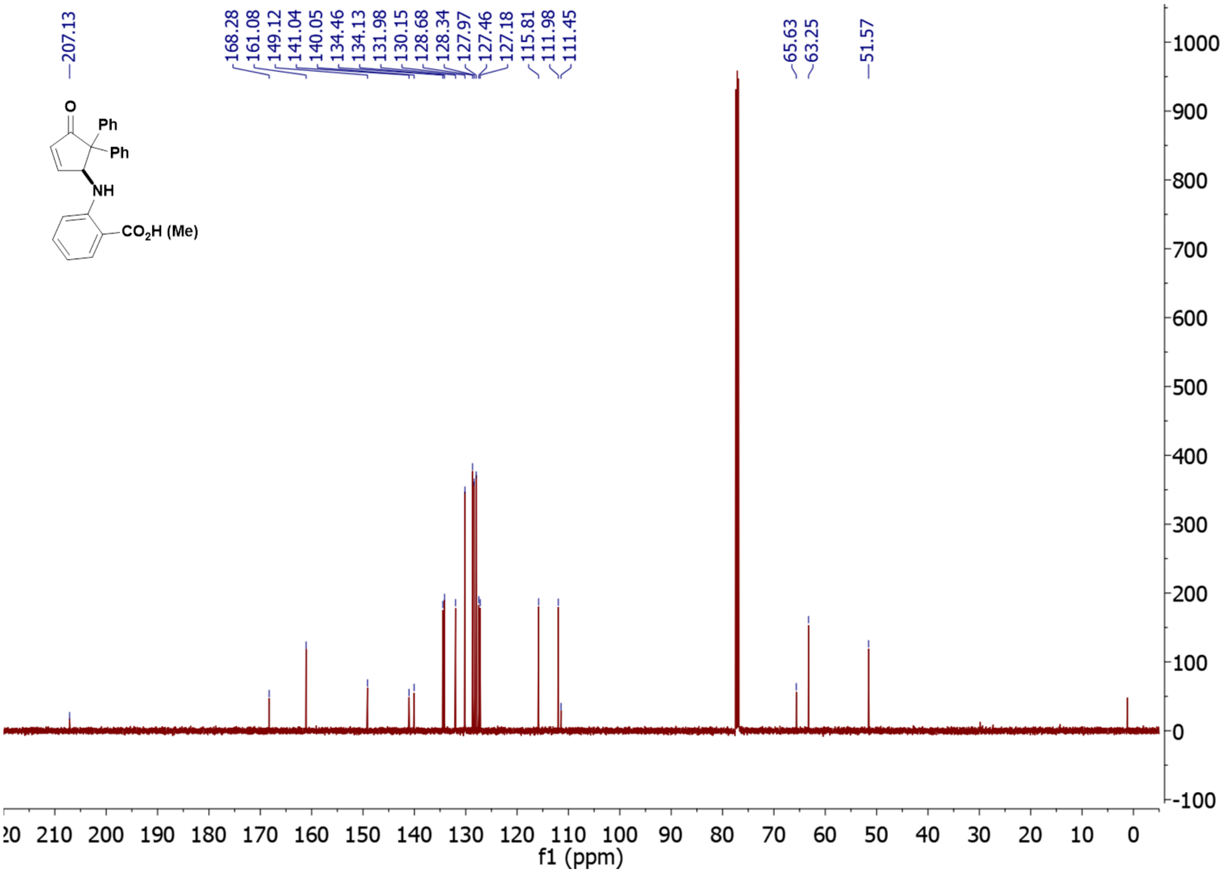

Supplement: File 1 — Experimental part and copies of NMR spectra. [file Beilstein_J_Org_Chem-15-1569-s001.pdf]
